# Effect of Bodily Fluids from Honey Bee (*Apis mellifera*) Larvae on Growth and Genome-Wide Transcriptional Response of the Causal Agent of American Foulbrood Disease (*Paenibacillus larvae*)

**DOI:** 10.1371/journal.pone.0089175

**Published:** 2014-02-20

**Authors:** Lina De Smet, Dieter De Koker, Alyse K. Hawley, Leonard J. Foster, Paul De Vos, Dirk C. de Graaf

**Affiliations:** 1 Ghent University, Laboratory of Zoophysiology, Department of Physiology, Ghent, Belgium; 2 University of British Columbia, Department of Microbiology & Immunology, Vancouver, Canada; 3 University of British Columbia, Department of Biochemistry & Molecular Biology, Vancouver, Canada; 4 Ghent University, Laboratory of Microbiology, Department of Biochemistry and Microbiology, Ghent, Belgium; Swedish University of Agricultural Sciences, Sweden

## Abstract

*Paenibacillus larvae*, the causal agent of American Foulbrood disease (AFB), affects honey bee health worldwide. The present study investigates the effect of bodily fluids from honey bee larvae on growth velocity and transcription for this Gram-positive, endospore-forming bacterium. It was observed that larval fluids accelerate the growth and lead to higher bacterial densities during stationary phase. The genome-wide transcriptional response of *in vitro* cultures of *P. larvae* to larval fluids was studied by microarray technology. Early responses of *P. larvae* to larval fluids are characterized by a general down-regulation of oligopeptide and sugar transporter genes, as well as by amino acid and carbohydrate metabolic genes, among others. Late responses are dominated by general down-regulation of sporulation genes and up-regulation of phage-related genes. A theoretical mechanism of carbon catabolite repression is discussed.

## Introduction

American Foulbrood (AFB) is a devastating brood disease affecting honey bee health worldwide [1]. The causal agent of AFB is the endospore-forming, Gram-positive bacterium *Paenibacillus larvae*. One-day-old bee larvae are most susceptible to infection [2], requiring less than ten orally ingested spores to establish a lethal infection [3]. After germination of ingested spores, vegetative *P. larvae* bacteria multiply within the gut lumen and subsequently breach the gut epithelium to reach the hemocoel [4]. Honey bee larvae are then degraded to brownish glue-like remains (ropy stage), which thereafter dries down to hard foulbrood scales containing approximately 2.5 billion spores [5].

In most countries AFB is a notifiable disease and major differences exist in imposed containment strategies. Until the late nineties, many countries followed an eradication procedure with destruction by burning. This drastic act seemed necessary for annihilation of the persistent and resistant spores [6], [7], until it was proven that large discrepancies exist between the number of outbreaks and the spread of spores [8]. Some countries allow administration of antibiotics. This practice, however, does not affect the infectious spore stage and leads to antibiotic resistance [9] and contamination in the honey [10]. More recently there is a tendency to control the disease by regular monitoring and elimination of symptomatic colonies. A fair amount of research focuses on alternative strategies [11], such as antagonistic bacteria [12], plant extracts [13], propolis [14], fatty acids [15], gamma radiation [16] and shook-swarm [17] treatments. The development of innovative treatment regimens will benefit from molecular knowledge on host-pathogen interactions during infection [18]. Such research is facilitated by the availability of the *P. larvae* genome sequence [19], [20] and it is within this conceptual framework that the present study was undertaken.

More specifically, this study investigates the growth velocity and genome-wide gene expression for *P. larvae*, by microarray technology, associated with *in vitro* exposure to bodily fluids from honey bee larvae. This approach was inspired by previous microarray studies reporting transcriptional changes for (pathogenic) bacteria in response to host material [21]–[23]. More precisely, microarray analysis was used to identify genes that were differentially expressed when potato extracts were added to the growth medium from *Pectobacterium atrosepticum* the causing agent of black leg disease in potato. Interestingly, some of the identified genes encoded virulence determinants.

Here we have examined (1) if honey bee larval bodily fluids could change *P. larvae* gene expression - e.g. overexpressing of genes encoding virulence factors - and (2) if these transcriptional changes correlated with altered *P. larvae* growth phenotypes.

## Materials and Methods

### Preparation of Honey Bee Larval Bodily Fluids

Larval bodily fluids were prepared by pooling third, fourth and fifth instars of the honey bee, *Apis mellifera carnica*, collected from the experimental beekeeping facility (Department of Physiology, Laboratory of Zoophysiology, University of Ghent, Belgium). Phenylthiourea (Hopkins & Williams Ltd) [100 µg per ml phosphate buffered saline (PBS, 20 mM KH_2_PO_4_, 60 mM Na_2_HPO_4_ and 145 mM NaCl, pH 7.2)], an inhibitor of phenoloxidase, was added immediately after squeezing the larvae to a final concentration of 10 µg/ml to prevent melanization. The homogenate was centrifuged twice for 30 min at 75,600×g and 4°C with an Avanti J30-I centrifuge (Beckman Coulter, Inc.). The clarified supernatant was filter sterilized with a 0.2 µm filter (Whatman) to render the bodily fluid for *P. larvae* culture spiking.

### *P. Larvae* Strain and Genotyping

*P. larvae* strain BRL-230010 was kindly provided by Dr. Queenie Chan (University of British Columbia, Canada). This was isolated from scales collected from a single severely diseased colony in Berkely, CA, USA. The strain was genotyped as described in [24].

### Effect of Larval Bodily Fluids on *P. Larvae* in vitro Growth

Throughout the experiments *P. larvae* strain BRL-230010 was routinely grown on brain heart infusion with thiamine (BHIT) broth at 37°C with agitation on an orbital shaker operating at 200 rpm [25]. Liquid bacterial cultures were started by inoculating BHIT broth with one *P. larvae* colony, grown for three days on BHIT agar at 37°C.

Examination of the potential (phenotypical) effect of host material on *P. larvae* growth (as a function of time) was first carried out by spiking bacterial cultures (in test tubes) at an OD_590_ of 0.2 with different concentrations of larval fluids. The same volume (1 ml) of different dilutions of larval bodily fluids was added to 1 ml culture to obtain respectively an 1/10, 1/25, 1/50, 1/100, 1/250 and 1/500 dilution. Each dilution was tested in triplicate. Addition of BHIT (with PTU in PBS) to liquid *P. larvae* cultures, instead of larval liquids, always served as a negative control. Addition of bodily fluids didn’t influence the absorbance at 590 nm.

Theoretical growth curves were modeled with DMFit [26]. Values for ‘rate’ and ‘yEnd’ were used as estimators for bacterial growth during exponential and stationary phase, respectively, and statistically analyzed with Kruskal-Wallis and Dunn’s test by GraphPad Prism (GraphPad Software, Inc.). The latter took only the comparisons for the 1/10 and 1/25 fluid dilution versus a control into account.

### Reference Gene Selection and Validation of Microarray Data by RT-qPCR

#### Growth and harvest of *P. larvae* cells

Reference gene stability was determined for 66 independent *P. larvae* 3 ml cultures (in test tubes), grown at the above-mentioned conditions, until they reached an OD_595_ of 0.2. At this density, 33 cultures were spiked with 4% (final concentration) larval bodily fluids (t, test) and 33 with BHIT broth (c, control). Eleven test cultures were further incubated for one hour (11×t1), eleven others for three hours (11×t3) and the remaining eleven for nine hours (11×t9). The same incubation regime was dictated to the control cultures (11×c1, 11×c3, 11×c9), thereby creating six different conditions (t1, t3, t9, c1, c3, c9). Bacterial cells were collected by centrifuging for 5 min at 8720×g and 4°C. The bacterial pellets were immediately resuspended in 650 µl RNA*later* Solution (Ambion). Aliquots (50 µl each) of these suspensions were incubated on ice for 30 min, centrifuged for 5 min at 6708×g and 4°C and stored at −20°C until RNA preparation.

#### RNA preparation

Prior to RNA extraction thawed aliquots were centrifuged for 2 min at 6708×g and 4°C. Bacterial pellets were resuspended by vortexing in 100 µl TE buffer (10 mM Tris-HCl, 1 mM EDTA, pH 8.0), containing lysozyme (15 mg/ml). The samples were incubated for 10 min at room temperature with constant shaking.

RNA was isolated with the InviTrap Spin Cell RNA Mini Kit (Invitek), using the protocol “Total RNA extraction from Gram-positive or Gram-negative bacteria” provided by the manufacturer. Additionally, an on-column DNase I treatment with the RNase-free DNase set (Qiagen) was performed. RNA was eluted in 40 µl elution buffer and stored at −20°C.

*cDNA synthesis*. RNA (5 µg) was converted to cDNA using random primers with the RevertAid First Strand cDNA Synthesis Kit (Fermentas), according to the manufacturer’s instructions.

#### Primer design and secondary structure formation of amplicon

Primers for nine candidate reference genes (Table S1 in File S1), with product size-ranges of 80 to 150 bp, were designed with Primer3Plus [27], using the default settings. Amplicon secondary structures were evaluated with MFold [28], using the default settings except for the folding temperature (60°C) and ionic concentrations ([Na^+^] = 50 mM, [Mg^2+^] = 3 mM).

#### RT-qPCR reaction mixture

For the RT-qPCR assay the reaction Platinum SYBR Green qPCR SuperMix-UDG (Invitrogen) kit was used. Each 15 µl reaction consisted of 7.5 µl SYBR master mix, 0.2 µM forward and 0.2 µM reverse primers (Integrated DNA Technologies) and 1 µl cDNA template using the CFX96 Real-Time PCR Detection System (Bio-Rad). The PCR program comprises a UDG digestion step of 2 min at 50°C, an activation step of 2 min at 95°C and 40 cycles of a combined denaturation (20 sec at 95°C) and annealing (40 sec at 60°C) step. At the end of this program a melt curve is generated by measuring fluorescence after each temperature increase of 0.5°C for 5 sec over a range from 65°C to 95°C. Primer efficiencies, R^2^ values and melt curves were calculated with CFX Manager Software (Bio-Rad). Reference gene stability was analyzed with the geNorm^PLUS^ algorithm within the qBase^PLUS^ environment (Biogazelle NV). Default settings were kept, except that target specific amplification efficiencies were used. Differential gene expression of twenty target genes (Table S2 in File S1) was statistically assessed with qBase^PLUS^ [29], [30], by means of unpaired t-tests (T4–C4 and T4–T1) or Mann-Whitney U tests (T1–C1 and C4–C1).

### Sample Preparation for Microarray and Validation Experiment

#### Growth and harvest of *P. larvae* cells

Samples for the microarray and the validation (by RT-qPCR) experiment were prepared by growing 16 independent 20 ml *P. larvae* cultures in 250 ml Erlenmeyer flasks, under the above mentioned conditions. The conditions were slightly changed in comparison with previous experiments in order to obtain enough bacterial cells to perform the microarray and validation experiment. Eight cultures were spiked with 5% (final concentration) larval bodily fluids (T, test) and eight with BHIT broth (C, control) at OD_595_ of 0.3. Four of the test and control cultures were incubated for 1 h (4× T1, and 4× C1) and the others for 4 h (4× T4, 4× C4). The bacterial cells were collected as described for reference gene selection and stored at −80°C until RNA preparation.

An accessory evaluation of *P. larvae* growth promotion in response to larval fluids was executed by comparing OD_590_ measurements on all bacterial cultures subjected to microarray analysis (16 samples: 4× C1, 4× T1, 4× C4, 4× T4). Comparisons T1–C1 and T4–C4 were statistically evaluated with a Mann-Whitney U test (p-value threshold = 0.05), using GraphPad Prism (GraphPad Software, Inc.).

#### RNA preparation

Bacterial pellets were resuspended in 200 µl TE buffer (30 mM Tris-HCl, 1 mM EDTA, pH 8.0) with additional proteinase K (Qiagen; 1/10 dilution). RNA was isolated with the RNeasy Plus Mini Kit, using the protocol “Purification of Total RNA for Animal Cells” and performing the optional on-column DNase I treatment, according to the manufacturer’s instructions. The RNA pellet was dissolved in 30 µl RNase-free water and stored at −80°C until use.

### Microarray Study on *P. Larvae* Transcriptional Response to Bee Bodily Fluids

#### Microarray experimental procedures

A custom 8×15 k Agilent array for *P. larvae* was developed with Agilent eArray software. RNA concentration and purity were determined spectrophotometrically using the Nanodrop ND-1000 (Nanodrop Technologies) and RNA integrity was assessed using a Bioanalyser 2100 (Agilent). For each sample, 5 µg of total RNA, spiked with 10 viral polyA transcript controls (Agilent), was converted to single stranded cDNA. The sample was subsequently labeled with Cyanine 3 (Cy3) mono-reactive dye or Cyanine 5 (Cy5) mono-reactive dye (GE Healthcare) according to the manufacturer’s protocol (two-color microarray-based prokaryote analysis (Fairplay III labeling) - Agilent). A mixture of purified and labeled cDNA (Cy3 label: 300 ng; Cy5 label: 300 ng) was hybridised followed by (manual) washing, according to the manufacturer’s procedures. To assess the raw probe signal intensities, arrays were scanned using the Agilent DNA MicroArray Scanner with SureScan High-Resolution Technology and probe signals were quantified using Agilent’s Feature Extraction software (version 10.7.3.1). The microarray data were deposited in the NCBI Gene Expression Omnibus (GEO) database (http://www.ncbi.nlm.nih.gov/geo/) under accession numbers GPL15243 (microarray including detailed annotation) and GSE37481.

#### Microarray data quality control and statistical analysis

Statistical data analysis was performed on the processed Cy3 and Cy5 intensities, as provided by the Feature Extraction Software version 10.7. Further analysis was performed in the R programming environment, in conjunction with the packages developed within the Bioconductor project (http://www.bioconductor.org; [31]. Differential expression between the conditions was assessed via the moderated t-statistic, described in Smyth, G. [32] and implemented in the Limma package of Bioconductor. This moderated t-statistic applies an empirical Bayesian strategy to compute the gene-wise residual standard deviations and thereby increases the power of the test, especially beneficial for smaller data sets. To control the false discovery rate, multiple testing correction was performed [33] and a significant result was defined where any probe had a corrected p-value below 0.05 and an absolute fold change larger than 2. As all probes had 3 or 4 replicates on the array, only those probes were retained that were called differentially expressed in 2 out of 3 or 3 out of 4. Genes were considered as (significantly) differentially expressed if (1) their BH-corrected p-value fell below 0.05, (2) their fold changes were at least 2 and (3) if at least 75% of the different probes for a same gene met the first two criteria.

### Microarray Data Functional Analysis

*P. larvae* genes were functionally annotated (already before qPCR validation) with Blast2GO [34], stand-alone BLAST [20] and KAAS (KEGG Automatic Annotation Server) [35] plus KEGG Mapper [36]. Putative transporter genes were also annotated with TransporterDB [37], putative transcription regulator genes with DBD [38] and RegPrecise [39] and putative proteolytic enzymes with MEROPS [40], [41]. COG functional categories were assigned with COGNITOR and stand-alone PSI-BLAST using the COG database [42]. After GO term annotation, an enrichment analysis (two-tailed Fisher’s exact test with default settings) within the Blast2GO environment was undertaken to compare different conditions.

#### Annotation and pathway pipeline

The annotation and pathway pipeline employed here finds ORFs with Prodigal [43], annotates genes with blastp against KEGG [44], COG [42], Metacyc [45] and RefSeq [46] and makes a Pathway Genome Database (PGDB) with Pathway Tools [47].

## Results

### Honey Bee Larval Bodily Fluid Stimulates in vitro Growth of *P. Larvae*

**Figure 1:**
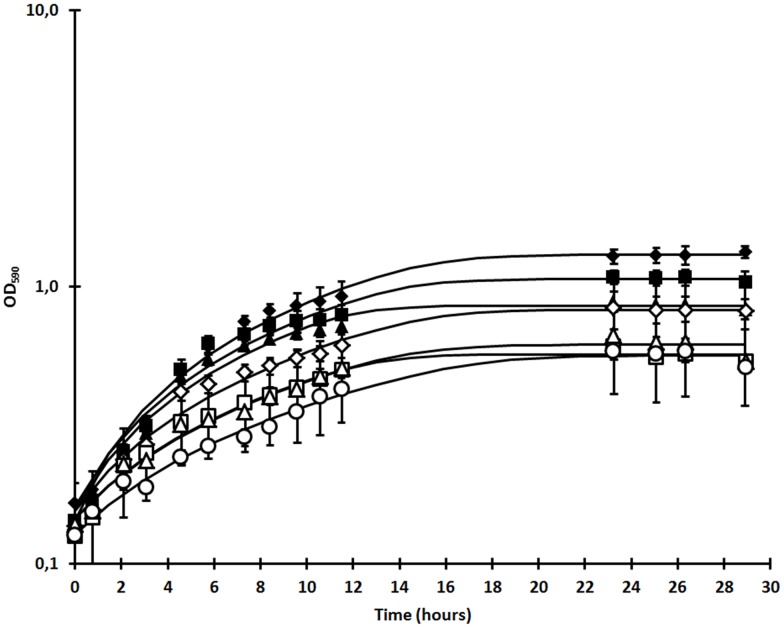
Effect of honey bee larval bodily fluid on *Paenibacillus larvae* growth. Effect of different concentrations of honey bee larval bodily fluid on the *in vitro* growth of *P. larvae* bacterial cells, expressed as the optical density measured at a wavelength of 590 nm (OD_590_) in function of time (hours). Growth alterations were determined for six bodily fluid concentrations, expressed as the fold dilution in BHIT broth cultures: 10× dilution (♦), 25× dilution (▪), 50× dilution (▴), 100× dilution (⋄), 250× dilution (□), 500× dilution (▵), control (○). Each point in the graph displays the mean of three independent replicates, with the error bars being the standard deviations. Time 0 represents the time of spiking. Trend lines are calculated with DMfit. No OD measurements were performed during lag phase.

**Figure 2:**
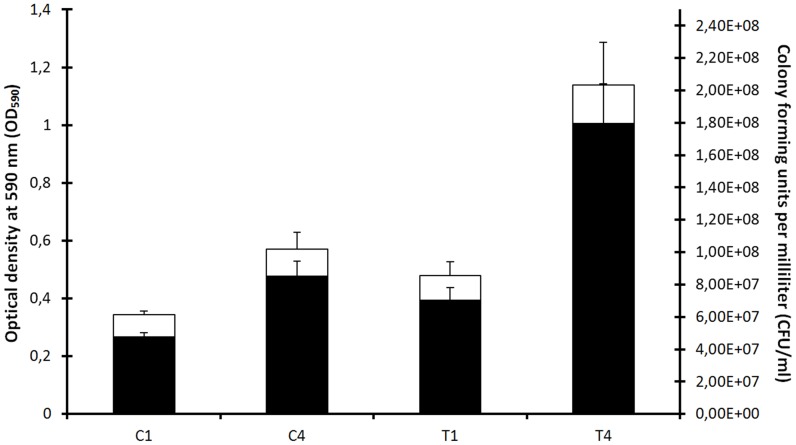
Effect of honey bee larval bodily fluid on *Paenibacillus larvae* bacterial density. OD590 (white bars) and CFU/ml (black bars) for T1, C1, T4 and C4. T1 (n = 12): test sample collected one hour after spiking with 5% larval fluids. T4 (n = 12): test sample collected four hours after spiking with 5% larval fluids. C1 (n = 11): control sample collected one hour after spiking with BHIT-broth. C4 (n = 12): control sample collected four hours after spiking with BHIT-broth. Between brackets (n): number of independent replicates. Error bars: standard deviations.

The *P. larvae* strain BRL 230010 was genotypes via REP-PCR using ERIC primers and could be assigned to ERIC I. We studied the effect of larval bodily fluids on *P. larvae* gene expression by *in vitro* exposure of bacterial cultures to larval fluids. We could show treatment-induced bacterial growth shifts (Figure 1). Merely considering the two highest fluid concentrations (in the *post hoc* analysis; 1/10 and 1/25 dilutions), both seem to significantly (p<0.05) promote *in vitro* growth of *P. larvae* cultures, in comparison to control cultures. In addition (significantly) higher bacterial densities are observed as a result of larval fluid treatment (Figure 2). Thus, it is now reasonable to assume that these bacterial growth shifts reflect the phenotypic response to altered transcription and so the subsequent microarray experiment was be performed using 5% larval fluids.

### Reference Gene Selection for Normalization of RT-qPCR Data

The reliability of the data of the microarray experiment was checked with RT-qPCR. To correct for experimental error qPCR data require normalization against reference genes [48]. To this end, 9 reference genes were selected (Table S1 in File S1) and their expression in *P. larvae* was quantified for six different *in vitro* growth conditions: one, three or nine hours after spiking with larval bodily fluid (test) or BHIT broth (control). Arranging the genes from most to least stably expressed across all conditions, produced the following ranking (Figure S1A in File S1): rpoD (0.631)<gyrA (0.634)<cmk (0.656)<sucB (0.694)<eftu (0.848)<fum (0.944) <purH (1.03)<adk (1.116)<gapdh (1.195). Between brackets the geNorm M-value, assessing gene stability, is indicated. Another measure, the geNorm V-value, is useful for determining the optimal number of reference genes for data normalization (Figure S1B in File S1): V2/3 (0.214) – V3/4 (0.157) – V4/5 (0.200) – V5/6 (0.166) – V6/7 (0.155) – V7/8 (0.151) – V8/9 (0.141). Setting the threshold to 0.15, all tested genes except *gapdh* should be included in the calculation of the normalization factors [30].

### Validation of Microarray Data by RT-qPCR

Microarray validation by RT-qPCR was performed with twenty randomly picked genes, belonging to different functional groups (Table S2 in File S1). Expression profiles obtained in the RT-qPCR experiment were similar in comparison to those of the microarray experiment, for all of the twenty genes in all of the four conditions (Figure S2 in File S1 and Figure S3 in File S1; Table S3 in File S1 and Table S4 in File S1).

### General Overview of Differential Gene Expression as Revealed by Microarray Analysis

Depicting the microarray data as a heatmap analysis allowed us to correlate the corrected Cy3 and Cy5 dye intensities showed separate clusters for all eight samples (four replicates, each labeled with both Cy3 and Cy5) of C1, T1 and C4 respectively, and for six of the eight samples of T4 (not shown). In order not to corrupt true biological patterns, one of the four samples for T4, clustering together with C1, was omitted from further analysis. Table 1 shows the total number up- and down-regulated genes for each of the four comparisons (T4–C4, T1–C1, T4–T1, C4–C1). The three selection criteria were p-value <0.05, fold change ≥2 and at least 75% of the different, hybridized probes for the same gene shows differential expression for a particular comparison.

**Table 1:** General overview of the microarray experiment, showing the total number of up- and down-regulated genes, for three selection criteria (columns) and four comparisons (rows).

	up-regulated			down-regulated		
	P<0.05 |FC| ≥2 Hyb ≥75%	P<0.05 |FC| ≥2	P<0.05	P<0.05 |FC| ≥2 Hyb ≥75%	P<0.05 |FC| ≥2	P<0.05
**T4–C4**	201	224	434	248	266	571
**T1–C1**	147	172	686	310	321	641
**T4–T1**	373	386	663	243	261	769
**C4–C1**	136	138	257	87	92	204

T1: test sample collected one hour after spiking with larval bodily fluid. T4: test sample collected four hours after spiking with larval bodily fluid. C1: control sample collected one hour after spiking with BHIT broth. C4: control sample collected four hours after spiking with BHIT broth. P: p-value. FC: fold change. Hyb: at least 75% of the different, hybridized probes for the same gene shows differential expression for a particular comparison.

### General Biological Patterns from Microarray Data by GO Term Assignment and Enrichment

At first GO terms were assigned to the *P. larvae* gene sequences. Using Blast2GO with Gene Ontology (GO) terms describing cellular component, biological process or molecular function, 2050 of the 3490 (unique) predicted genes within the *P. larvae* genome were assigned to at least one GO category. Subsequently GO term enrichment analysis identified (significantly) over- or underrepresented GO terms for the differentially expressed genes for each of the four comparisons (Table 2, Table 3, Table S5 in File S1 and Table S6 in File S1). The annotation and pathway pipeline annotated 10/147, 15/210, 9/136 and 45/373 of the up-regulated and 34/310, 19/248, 7/87 and 14/243 of the down-regulated genes for T1–C1, T4–C4, C4–C1 and T4–T1 (Table 4, Table 5, Table S7 in File S1 and Table S8 in File S1).

**Table 2:** GO-enrichment analysis for comparison T1–C1, showing the most specific over- and under-represented biological process GO-terms for both up- and down-regulation.

Biological process	GO number	NS	FE	P-value
**Up-regulation**				
**Over-representation**				
Electron transport chain	GO:0022900	6	13.49	3.70E-05
Nitrate assimilation	GO:0042128	3	∞	6.90E-05
**Down-regulation**				
**Over-representation**				
Benzoate metabolic process	GO:0018874	6	12.73	2.10E-04
β-alanine metabolic process	GO:0019482	5	14.09	5.90E-04
Carbohydrate catabolic process	GO:0016052	15	3.18	5.50E-04
Carboxylic acid biosynthetic process	GO:0046394	27	2.11	1.70E-03
Electron transport	GO:0006118	13	3.03	1.70E-03
Inositol metabolic process	GO:0006020	6	6.35	2.10E-03
Limonene catabolic process	GO:0046251	5	∞	1.40E-05
Lysine catabolic process	GO:0006554	5	∞	1.40E-05
Mitochondrial electron transport, NADH to ubiquinone	GO:0006120	5	∞	1.40E-05
Oligopeptide transport	GO:0006857	4	∞	1.30E-04
PEP-dependent sugar PTS	GO:0009401	9	5.13	5.10E-04
Photosynthesis, light reaction	GO:0019684	5	∞	1.40E-05
Reductive tricarboxylic acid cycle	GO:0019643	6	6.35	2.10E-03
Sodium ion transport	GO:0006814	7	11.93	7.60E-05
Sulfate transport	GO:0008272	3	∞	1.20E-03
Sulfur amino acid metabolic process	GO:0000096	10	3.56	2.20E-03
Ubiquinone biosynthetic process	GO:0006744	5	21.15	2.40E-04
**Under-representation**				
DNA metabolic process	GO:0006259	0	0	1.80E-08
RNA metabolic process	GO:0016070	8	0.34	1.20E-03
Translation	GO:0006412	0	0	4.00E-05

NS: number of differentially expressed genes, annotated with a particular GO-term. FE: fold enrichment (>1: over-represented GO terms; <1: under-represented GO terms; 0: zero genes in the test group, annotated with a particular GO term; ∞: zero genes in the reference group, annotated with a particular GO term). FE for a particular GO term is calculated as follows: (number of genes with GO term in test group/number of genes with GO term in reference group)/(number of genes without GO term in test group/number of genes without GO term in reference group). P-value: significance measure (cut-off: 0.05) as calculated by Blast2GO during enrichment analysis.

**Table 3:** GO-enrichment analysis for comparison T4–C4, showing the most specific over- and under-represented biological process GO-terms for both up- and down-regulation.

Biological process	GO number	NS	FE	P-value
**Up-regulation**				
**Over-representation**				
Biotin biosynthetic process	GO:0009102	4	∞	6.00E-06
Electron transport chain	GO:0022900	6	10.89	1.10E-04
Glyoxylate cycle	GO:0006097	3	∞	1.20E-04
Nitrate assimilation	GO:0042128	3	∞	1.20E-04
Pyrimidine nucleotide biosynthetic process	GO:0006221	5	10.99	4.00E-04
Reductive tricarboxylic acid cycle	GO:0019643	5	10.99	4.00E-04
**Under-representation**				
Cellular macromolecule metabolic process	GO:0044260	9	0.26	8.90E-06
Nucleic acid metabolic process	GO:0090304	8	0.33	8.70E-04
**Over-representation**				
Benzoate metabolic process	GO:0018874	5	14.34	2.50E-04
β-alanine metabolic process	GO:0019482	4	14.24	1.10E-03
High-affinity iron ion transport	GO:0006827	4	∞	2.00E-05
Isoleucine catabolic process	GO:0006550	5	11.94	4.30E-04
Leucine catabolic process	GO:0006552	5	11.94	4.30E-04
Limonene catabolic process	GO:0046251	4	57.04	9.30E-05
Lysine catabolic process	GO:0006554	4	57.04	9.30E-05
Oligopeptide transport	GO:0006857	3	42.47	1.10E-03
Potassium ion transport	GO:0006813	5	35.90	2.40E-05
Siderophore biosynthetic process	GO:0019290	4	∞	2.00E-05
Sporulation resulting in formation of a cellular spore	GO:0030435	7	12.72	2.20E-05
Sulfate transport	GO:0008272	3	∞	3.00E-04
Valine catabolic process	GO:0006574	5	11.94	4.30E-04
**Under-representation**				
Cellular macromolecule metabolic process	GO:0044260	18	0.40	1.30E-04
Nucleobase, nucleoside, nucleotide and nucleic acid metabolic process	GO:0006139	20	0.47	1.60E-03

NS, FE, P-value: see Table 2.

**Table 4:** Annotation and pathway analysis for both up- and down-regulated genes for T1–C1.

Pathway	ID	#
**Up-regulation**		
Alkylnitronates degradation	PWY-723	3/4
Arginine degradation III	PWY0-823	1/2
Arginine dependent acid resistance	PWY0-1299	1/1
Asparagine degradation I	ASPARAGINE-DEG1-PWY	1/1
Demethylmenaquinol-8 biosynthesis I	PWY-5852	1/1
Leucine biosynthesis	LEUSYN-PWY	1/7
Pyruvate fermentation to ethanol I	PWY-5480	1/4
Reductive monocarboxylic acid cycle	PWY-5493	1/3
Tetrapyrrole biosynthesis I	PWY-5188	1/7
Trehalose degradation I	TREDEGLOW-PWY	1/4
**Down-regulation**		
5-dehydro-4-deoxy-D-glucuronate degradation	PWY-6507	2/4
Acetate conversion to acetyl-CoA	PWY0-1313	1/1
Acetyl-CoA biosynthesis	PYRUVDEHYD-PWY	3/9
Adenine and adenosine salvage IV	PWY-6610	1/5
Arginine degradation VI	ARG-PRO-PWY	1/4
Arginine degradation VII	ARG-GLU-PWY	2/2
Aspartate degradation II	MALATE-ASPARTATE-SHUTTLE-PWY	1/2
Branched-chain alpha-keto acid dehydrogenase complex	PWY-5046	1/6
Citrulline biosynthesis	CITRULBIO-PWY	2/9
Cysteine biosynthesis I	CYSTSYN-PWY	2/5
DIMBOA-glucoside degradation	PWY-4441	1/1
dTDP-L-rhamnose biosynthesis I	DTDPRHAMSYN-PWY	2/7
Ethanol degradation II	PWY66-21	1/4
Ethanol degradation IV	PWY66-162	2/4
Glutamate biosynthesis I	GLUTSYN-PWY	1/1
Glutamate biosynthesis IV	GLUGLNSYN-PWY	1/1
Glutamine degradation II	GLUTAMINEFUM-PWY	1/1
Glycine betaine biosynthesis II	PWY-3722	1/2
Glycine cleavage complex	GLYCLEAV-PWY	1/6
Guanine and guanosine salvage I	PWY-6620	1/4
Lysine biosynthesis III	PWY-2942	1/7
Lysine biosynthesis VI	PWY-5097	3/9
Methionine biosynthesis I	HOMOSER-METSYN-PWY	1/7
NADH to cytochrome bd oxidase electron transfer	PWY0-1334	10/11
Ornithine degradation I	ORN-AMINOPENTANOATE-CAT-PWY	1/1
Selenocysteine biosynthesis I	PWY0-901	1/4
Superoxide radicals degradation	DETOX1-PWY	1/4
Xanthine and xanthosine salvage	SALVPURINE2-PWY	1/3

ID: MetaCyc identifier. #: number of up- or down-regulated genes annotated with a particular pathway in relation to the number of genes annotated with that pathway.

**Table 5:** Annotation and pathway analysis for both up- and down-regulated genes for T4-C4.

Pathway	ID	
**Up-regulation**		
Alkylnitronates degradation	PWY-723	3/4
Aspartate degradation II	MALATE-ASPARTATE-SHUTTLE-PWY	1/2
Biotin biosynthesis from 7-keto-8-aminopelargonate	PWY0-1507	4/4
Cadmium transport I	PWY-6213	1/2
Folate polyglutamylation	PWY-2161	1/3
Formate oxidation to CO2	PWY-1881	1/1
Glutamate biosynthesis I	GLUTSYN-PWY	1/1
Glutamate biosynthesis IV	GLUGLNSYN-PWY	1/1
Glutamine degradation II	GLUTAMINEFUM-PWY	1/1
Glycine biosynthesis I	GLYSYN-PWY	1/1
Pentose phosphate pathway (non-oxidative branch)	NONOXIPENT-PWY	1/5
Pyruvate fermentation to ethanol I	PWY-5480	1/4
Pyruvate fermentation to ethanol III	PWY-6587	1/5
Reductive monocarboxylic acid cycle	PWY-5493	2/3
**Down-regulation**		
2,3-dihydroxybenzoate biosynthesis	PWY-5901	3/3
Acetyl-CoA biosynthesis	PYRUVDEHYD-PWY	3/9
Adenine and adenosine salvage IV	PWY-6610	1/5
Branched-chain alpha-keto acid dehydrogenase complex	PWY-5046	1/6
Citrulline biosynthesis	CITRULBIO-PWY	1/9
Ethanol degradation IV	PWY66-162	1/4
Glycine cleavage complex	GLYCLEAV-PWY	1/6
Glycogen biosynthesis I	GLYCOGENSYNTH-PWY	5/7
Guanine and guanosine salvage I	PWY-6620	1/4
Isoleucine biosynthesis I	ILEUSYN-PWY	1/11
Leucine biosynthesis	LEUSYN-PWY	1/7
Methylthiopropionate biosynthesis	PWY-5389	1/1
NADH to cytochrome bd oxidase electron transfer	PWY0-1334	3/11
Starch biosynthesis	PWY-622	5/9
Superoxide radicals degradation	DETOX1-PWY	1/4
Valine biosynthesis	VALSYN-PWY	1/7
Xanthine and xanthosine salvage	SALVPURINE2-PWY	1/3

ID: MetaCyc identifier. #: number of up- or down-regulated genes annotated with a particular pathway in relation to the number of genes annotated with that pathway.

**Figure 3:**
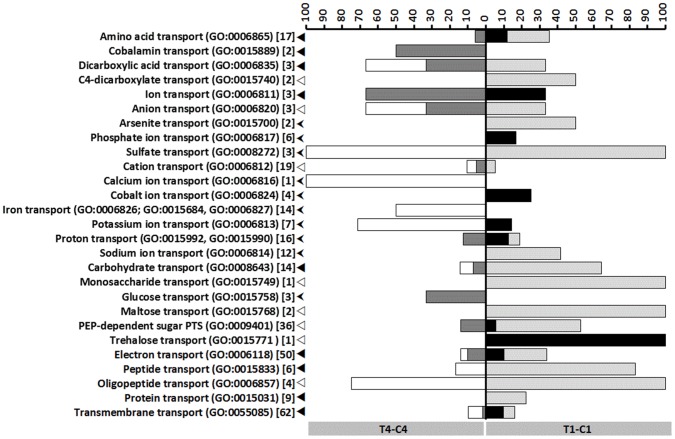
Differential expression of transporter encoding genes. Stacked percentage bar chart, showing the numbers of up- and down-regulated (putative) transporter encoding genes for T4-C4 (left) and T1-C1 (right), respectively, relative to the total numbers of (putative) transporter encoding genes within the *P. larvae* genome. The latter are indicated between square brackets. Round brackets: GO term numbers. GO terms were assigned with Blast2GO. White bars: down-regulation for T4-C4. Dark grey bars: up-regulation for T4-C4. Light grey bars: down-regulation for T1-C1. Black bars: up-regulation for T1-C1. Arrow heads: arbitrary GO term hierarchy (◂>⊲><$>\raster(90%)="rg3"<$>).

**Figure 4:**
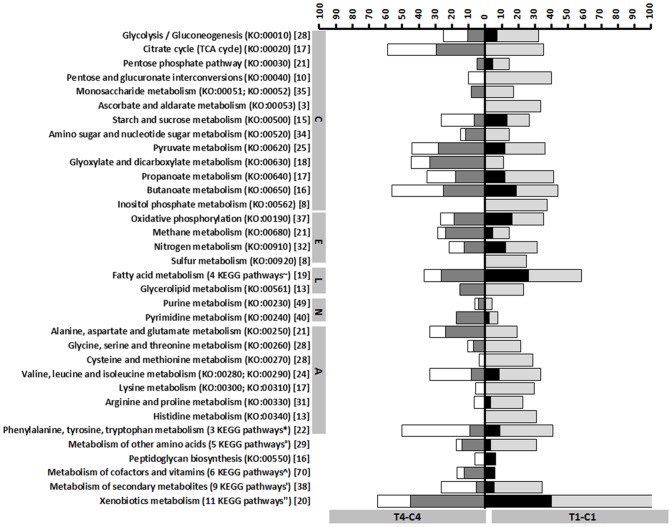
Differential expression of metabolic genes. Stacked percentage bar chart, showing the numbers of up- and down-regulated (putative) metabolic genes for T4-C4 (left) and T1-C1 (right), respectively, relative to the total numbers of (putative) metabolic genes within the *P. larvae* genome. C: carbohydrate metabolism. The latter are indicated between square brackets. Round brackets: KO numbers (KEGG pathways). KEGG pathways were assigned with KAAS. White bars: down-regulation for T4-C4. Dark grey bars: up-regulation for T4-C4. Light grey bars: down-regulation for T1-C1. Black bars: up-regulation for T1-C1. E: energy metabolism. L: lipid metabolism. N: nucleotide metabolism. A: amino acid metabolism. ∼: KO:00061; KO:00071; KO:00592; KO:01040. *: KO:00360; KO:00350; KO:00380. °: KO:00410; KO:00430; KO:00450; KO:00460; KO:00480. ?: KO:00740; KO:00770; KO:00780; KO:00670; KO:00860; KO:00130. ‘:KO:00900; KO:00903; KO:00281; KO:00523; KO:01053; KO:01055; KO:00940; KO:00311; KO:00521. ‘’: KO:00362; KO:00627; KO:00625; KO:00622; KO:00633; KO:00642; KO:00643; KO:00930; KO:00363; KO:00621; KO:00626.

**Figure 5:**
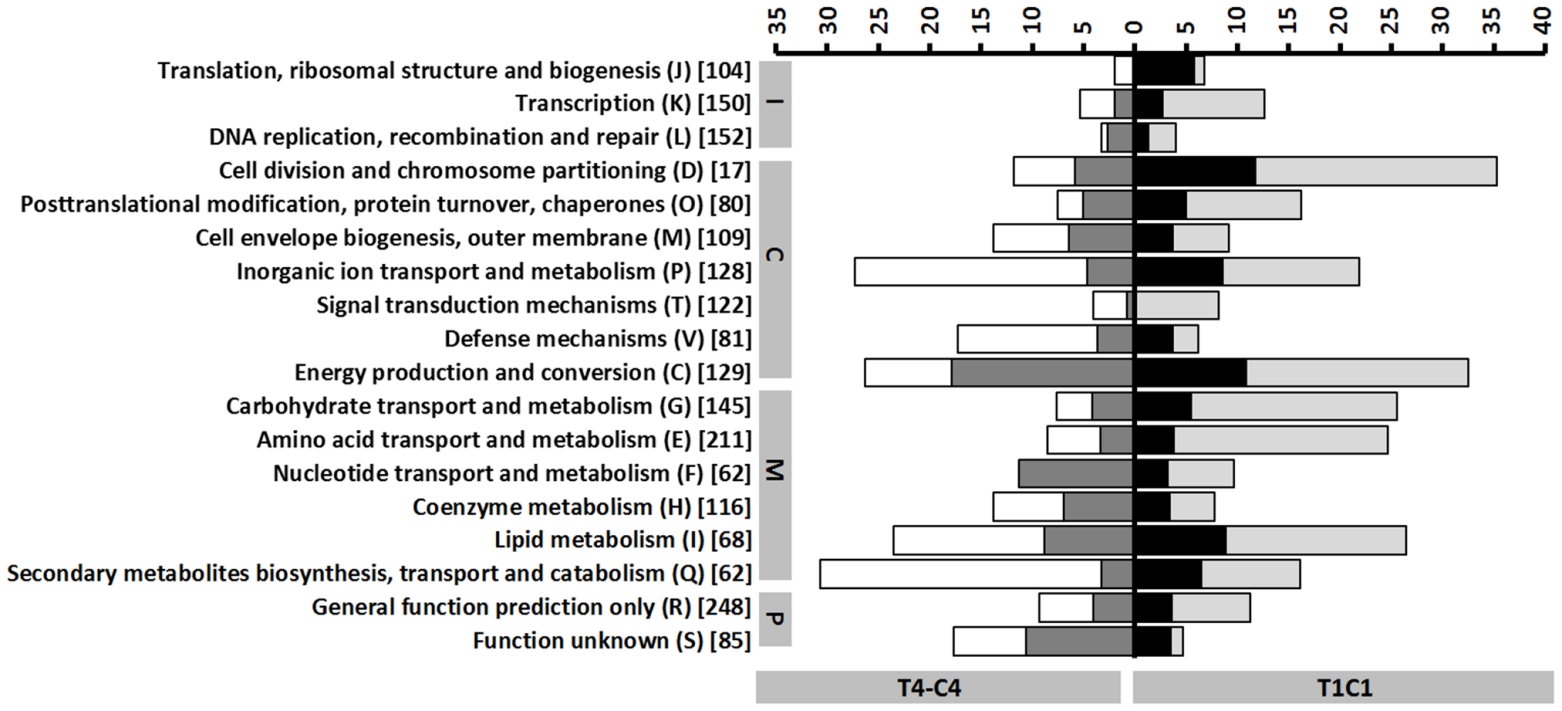
Numbers of genes that are differentially expressed per subset. Stacked percentage bar chart, showing the numbers of up- and down-regulated genes for T4-C4 (left) and T1-C1 (right), respectively, relative to the total numbers of genes within the *P. larvae* genome. The latter are indicated between square brackets. Round brackets: COG functional category label. C: cellular processes and signaling. I: information storage and processing. M: metabolism. P: poorly characterized. White bars: down-regulation for T4-C4. Dark grey bars: up-regulation for T4-C4. Light grey bars: down-regulation for T1-C1. Black bars: up-regulation for T1-C1.

The differently expressed transporter and metabolic genes are represented in stacked percentage bar chart in Figure 3 and Figure 4 for T4-C4 and T1-C1 and Figure S4 in File S1 and Figure S5 in File S1 for T4-T1 and C4-C1 General trends in biological processes are also graphically depicted, according to the COG (Clusters of Orthologous Groups) functional classification (Figure 5 and Figure S6 in File S1).

In control cultures (C1 and C4) *P. larvae* seems to invest more in oligopeptide and ion (sulfate ion; cation: Na^+^ for C1, K^+^ for C4) transport, amino acid (β-A, K; sulfur AAs for C1, BCAAs for C4), benzoate and limonene metabolism, while restricting nucleic acid metabolism. One hour after spiking (C1) *P. larvae* appears to rely more on carbohydrate (phosphotransferase system, inositol), carboxylic acid and ubiquinone metabolism, while limiting translation. The data also suggest that energy storage (electron transport, NADH to ubiquinone) and carbon fixation (photosynthesis, reductive citric acid cycle) are also upregulated. Four hours after spiking (C4) *P. larvae* tend to rely more on iron uptake (high-affinity iron transport and siderophore biosynthesis) and sporulation.

In test cultures, on the other hand, *P. larvae* seems to change its metabolism to nitrate assimilation and store energy through the electron transport chain. Four hours after spiking (T4) *P. larvae* appears to rely more on biotin, pyrimidine and carbon (TCA/glyoxylate cycle) metabolism. In addition, at late time points (T4-C4) *P. larvae* tend to restrict cellular macromolecular metabolic processes.

## Discussion

The reasons behind the trends observed in the gene expression data presented here become clear if one considers the chemical composition of bodily fluids from honey bee worker larvae. These fluids are thought to contain mainly hemolymph and liquid gut contents. Since this material was collected from third, fourth and fifth larval instars, the gut is thought to hold (digested) honey/nectar, pollen and royal jelly [49]. Honey/nectar is particularly rich in sugars (especially fructose and glucose; [50], while royal jelly is a source of proteins [51]. The protein composition from honey bee worker larvae has been described in most detail [52]. Paralleling the diet, hemolymph of adult worker bees is rich in sugars [53], free amino acids [54] and proteins [55]. Fresh [56] and powdered [57] bee brood has been shown to be especially rich in amino acids/proteins.

The general down-regulation of carbohydrate and amino acid/peptide metabolism and transport in the presence of larval fluids at early time points (T1-C1) is unexpected, although genes involved in trehalose metabolism and transport are strongly upregulated: phosphotrehalase (*treA*; 12.93×↑) and trehalose-specific IIBC subunit of PTS system (*treP*; 16.85×↑). This could point towards carbon catabolite repression (CCR): a phenomenon by which usage of secondary carbon sources is reduced in the presence of the preferred one, which allows the fastest growth [58]. Thus trehalose, the principle blood sugar of many insects [59], is probably the preferred sugar of *P. larvae*. This hypothesis is supported by observations made for the *Paenibacillus popilliae* [60]. This pathogen of beetle larvae takes up trehalose through the PTS system and phosphorylates it to trehalose 6-phosphate (T6 P) in a PEP-dependent way. T6 P is subsequently cleaved to glucose and glucose-6-phosphate (G6 P). Down-regulation of genes for citric acid cycle enzymes (T1-C1; *citB*: 1.32×↓ (ns); *citC*: 4.53×↓; *citH*: 3.64×↓; *citZ*: 8.39×↓; [61]) and virulence factors [62] - exemplified by toxin Etx/Mtx (T1-C1; >8.50×↓) - provide additional support to the CCR hypothesis. Genes *ccpA* and *codY*, however, are not differentially expressed. Their products are global regulators of carbon (glucose) metabolism by sensing FBP and G6P (for CcpA) and GTP and BCAA (for CodY) in *Bacillus subtilis* [61]. Additionally, fatty acid catabolism in *B. subtilis* is repressed through CCR [63]. In contrast, this study reveals a strong up-regulation for genes involved in fatty acid degradation (*eft*A, B: >24.50×↑; *fad*A, E, F, N, R: >20↑; *lcf*A: 14.51×↑).

The down-regulation of many oligopeptide permeases (opp) might reflect the high free amino acid content of insect hemolymph [64]. The investment of *P. larvae* in peptide transport, subsequent catabolism and amino acid synthesis will be minimal as amino acids are copiously provided. Sulphate transport is also repressed when cysteine is present [65] and because of the high free amino acid availability, the down-regulation of sulphate transporters (T1-C1; *cys*A, P, U, W; >40×↓) can be explained.

The up-regulated import of especially mannose (*lev*D, E, F, G; >5×↑/>11×↑) at later time points (T4-C4/T4-T1) indicate that the consumption of trehalose proceeds and *P. larvae* seems to switch to alternative carbon sources at higher cell densities. Although high densities are reached at T4 (OD_590_ ≈ 1.14), no starvation seems to occur (e.g. *ctsA*: >2.3×↓) and as a consequence sporulation is not initiated. Sporulation starts at late time points (C4-C1), which is suggested by the up-regulation of 19 from the 52 spore/sporulation related genes in the control cultures. Addition of larval bodily fluids delays sporulation initiation, which is illustrated by the down regulation of 35/52 sporulation related genes in the treated cultures; none of these genes showed up-regulation. Moreover, sporulation is mostly regulated by environmental signals through chemotaxis [66]. The gene expression patterns of putative signaling proteins (e.g. MCPs, HPKs, RRs) are similar to those of the sporulation related genes. Nutrient limitation and competition often leads to the production of antibiotics [67], which is reflected in the up-regulation of (putative) synthetases for antimicrobial biosurfactans, plipastatin (five genes) and surfactin (four genes) at later time points (T4-T1; C4-C1) however addition of larval bodily fluids seems to diminish the production and transport of antibiotics (T4-C4; T1-C1). Other antibiotic-related genes - amongst which penicillin acylase [68], two peptides AS-48 [69], four lantibiotic/lanthionine synthesis proteins [70], neotrehalosadiamine [71] and circularin A/uberolysin [72] - together show a similar pattern (with some exceptions). The same holds more or less for polyketide biosynthesis genes (e.g. *rfbB, C, D* of the rhamnose pathway; [73]). Closticin 574 tends to be up-regulated by larval fluids at late time points (T4-C4, T4-T1) [74].

Differential expression of (putative) cofactor and vitamin metabolic genes was observed for different pathways but one of the most up-regulated pathways in the presence of larval fluids at late time points is the one responsible for thiamine biosynthesis (e.g. *apb*E: 85.80×↑ for T4-C1 and 54.08×↑ for T4-T1; [75]). The same pathway is down-regulated in control cultures at late time points (C4-C1). This indicates that thiamine seems exhausted and biosynthesis of this vitamin/cofactor seems necessary. Another remarkable fact is the quite large-scale phage-related gene expression at late time points in the presence of larval fluids. If bacteriophage particles are produced, this might reflect spontaneous induction of the prophages’ lytic cycle through signal molecules of bacterial quorum sensing [76].

At late time points, oxygen appears to become limited. As a consequence *P. larvae* seems to switch from aerobic to anaerobic respiration/fermentation. Many bacteria follow a mixed acid fermentation route for glucose metabolism with end products ethanol, succinate, lactate, acetate, formate, and carbon dioxide [77]. Typical indicators of this process arise from the activity of its key enzyme, pyruvate formate lyase (Pfl), which leads to massive excretion of formate and acetate as fermentative by-products. Down-regulation of pyruvate dehydrogenase (*pdhA*: 5.39×↓ and *pdhB*: 5.01×↓) and up-regulation of pyruvate formate lyase (*pflA*: 21.61×↑, *pflB*: 9.56×↑) confirm this hypothesis. Moreover *P. larvae* seems to rely mostly on cytochrome *aa*3-600 quinol oxidase (*qoxA, B, C, D*: >9×↑; [78]) at T1-C1, while it switches to cytochrome *bd* ubiquinol oxidase (*cydA*: 76.47×↑, *cydB*: 37.08×↑) at T4-C4. The latter enzyme is expressed under oxygen-limiting conditions in *E. coli* [79]. Furthermore nitrogen metabolism seems boosted at T4-C4: formate/nitrite transport (*yrhG*: >3×↑), assimilatory nitrite reductase (*nasD, E*: >10×↑) and anaerobic regulator (*fnr*: 11.89×up) are all up-regulated. NasDE nitrite reductase functions as both an assimilatory and a dissimilatory enzyme which is produced under oxygen limiting and nitrogen limiting conditions [80]. The bacterial nitrogen cycle is complicated and still a matter of debate as different pathways are involved such as the dissimilative nitrate reduction to ammonia and the denitrification pathway with reduction of nitrate to N_2_. On the other hand, homologues to bifunctional alcohol/acetaldehyde dehydrogenase (*adh*E) are highly up-regulated at T4-C4 (108.91×↑, 97.52×↑ and 60.68× respectively↑). This could point towards sugar fermentation, converting acetyl-coA to acetaldehyde and then to ethanol. It has been suggested for *E. coli* that transcription of *adh*E is repressed through CCR under aerobic conditions (T1-C1; ±2×↑) and that expression of *adh*E is much higher in anaerobically grown cells [81].

Some other remarkable differences in gene expression as a result of the addition of larval bodily fluids are three differentially expressed (putative) toxin encoding genes. Two homologues of ε-toxins (ETX) or mosquitocidal toxins (MTX) [82] are down-regulated for T1-C1 (8.53×↓, 8.75×↓) and C4-C1 (2.56×↓, 2.60×↓), and up-regulated for T4-T1 (4.47×↑, 4.47×↑). The ι-toxin Ib component [83], on the contrary, is up-regulated for both T1-C1 (2.62×↑) and C4-C1 (2.06×↑). Next to toxins, proteases/peptidases have been proposed as putative *P. larvae* virulence factors [20], [62], [84], [85]. However, no general pattern emerges from the expression data which can be explained by their divers biological roles. Recently S-layer proteins have been suggested as potential *P. larvae* virulence determinants [86], [87]. Our study revealed up-regulation (±2.5×↑) of three such genes at late time points (T4–T1). At late time points the presence of larval bodily fluids favors motility which is reflected by the up-regulation of 7/8 genes. The eight differentially expressed (putative) flagellar genes (MC) encode (1) filament capping-protein (*fliD*), (2) filament (*fliC)*, (3) filament-hook junction (*flgK*, *flgL*), (4) basal body stator (*motA*, *motB*), (5) a flagellar export chaperone for FliC (*fliS*) and (6) others (*yvyF*) [88].

## Conclusions

We can conclude that the honey bee larval bodily fluid stimulates *in vitro* growth of *P. larvae* which is reflected by the large amount of transcriptional changes. Early responses are characterized by a general down-regulation of transporter genes and genes involved in the amino acid and carbohydrate metabolism. At later time points the sporulation genes are down-regulated while phage-related genes are up-regulated. The importance of the changed expression of phage-related genes will be a subject for further research.

## Supporting Information

File S1 **Figure S1.** Average expression stability (A) and determination of the optimal number (B) of reference targets with geNorm^PLUS^. **Figure S2.** Validation of microarray data with RTQ-PCR. (A) Log_2_-transformed expression ratio of T1 compared to C1. (B) Log_2_-transformed expression ratio of T4 compared to C4. White bars: RTQ-PCR experiment. Black bars: microarray experiment. T1: test sample collected one hour after spiking with larval fluids. T4: test sample collected four hours after spiking with larval fluids. C1: control sample collected one hour after spiking with BHIT-broth. C4: control sample collected four hours after spiking with BHIT-broth. +: differential expression (significant). -: equal expression (non-significant). **Figure S3.** Validation of microarray data with RTQ-PCR. (A) Log_2_-transformed expression ratio of C4 compared to C1. (B) Log_2_-transformed expression ratio of T4 compared to T1. White bars: RTQ-PCR experiment. Black bars: microarray experiment. T1: test sample collected one hour after spiking with hemolymph. T4: test sample collected four hours after spiking with hemolymph. C1: control sample collected one hour after spiking with BHIT-broth. C4: control sample collected four hours after spiking with BHIT-broth. +: differential expression (significant). -: equal expression (non-significant). **Figure S4.** Stacked percentage bar chart, showing the numbers of up- and down-regulated (putative) transporter encoding genes for T4–T1 (left) and C4–C1 (right), respectively, relative to the total numbers of (putative) transporter encoding genes within the *P. larvae* genome. The latter are indicated between square brackets. Round brackets: GO term numbers. GO terms were assigned with Blast2GO. White bars: down-regulation for T4–T1. Dark grey bars: up-regulation for T4–T1. Light grey bars: down-regulation for C4–C1. Black bars: up-regulation for C4–C1. Arrow heads: arbitrary GO term hierarchy (;◂>⊲><$>\raster="rg3"<$>). **Figure S5.** Stacked percentage bar chart, showing the numbers of up- and down-regulated (putative) metabolic genes for T4–T1 (left) and C4–C1 (right), respectively, relative to the total numbers of (putative) metabolic genes within the *P. larvae* genome. C: carbohydrate metabolism. The latter are indicated between square brackets. Round brackets: KO numbers (KEGG pathways). KEGG pathways were assigned with KAAS. White bars: down-regulation for T4–T1. Dark grey bars: up-regulation for T4–T1. Light grey bars: down-regulation for C4–C1. Black bars: up-regulation for C4–C1. ∼: KO:00061; KO:00071; KO:00592. *: KO:00350; KO:00360; KO:00380; KO:00400. °: KO:00410; KO:00430; KO:00450; KO:00480. ?: KO:00740; KO:00760; KO:00770; KO:00780; KO:00790; KO:00670; KO:00860; KO:00130. ‘:KO:00900; KO:00903; KO:00281; KO:00523; KO:01053; KO:01055; KO:00960; KO:00232; KO:00521; KO:00401. ‘’: KO:00362; KO:00627; KO:00625; KO:00622; KO:00633; KO:00642; KO:00643; KO:00930; KO:00621; KO:00626; KO:00983. **Figure S6.** Stacked percentage bar chart, showing the numbers of up- and down-regulated genes for T4–T1 (left) and C4–C1 (right), respectively, relative to the total numbers of genes within the *P. larvae* genome. The latter are indicated between square brackets. Round brackets: COG functional category label. C: cellular processes and signaling. I: information storage and processing. M: metabolism. P: poorly characterized. White bars: down-regulation for T4–T1. Dark grey bars: up-regulation for T4–T1. Light grey bars: down-regulation for C4–C1. Black bars: up-regulation for C4–C1. **Table S1.** Reference genes used to normalize the results of the qRT-PCR experiment for microarray data validation. **Table S2.** Randomly selected genes used in the RTQ-PCR experiment to validate the microarray results. **Table S3.** Validation of microarray data by qRT-PCR. **Table S4.** Validation of microarray data by qRT-PCR. **Table S5.** GO-enrichment analysis for comparison C4–C1, showing the most specific over- and under-represented biological process GO-terms for both up- and down-regulation. **Table S6.** GO-enrichment analysis for comparison T4–T1, showing the most specific over- and under-represented biological process GO-terms for both up- and down-regulation. **Table S7.** Annotation and pathway analysis for both up- and down-regulated genes for C4–C1. **Table S8.** Annotation and pathway analysis for both up- and down-regulated genes for T4–T1.
(PDF)
