# Supplementary material for: Effect of Bodily Fluids from Honey Bee (Apis mellifera) Larvae on Growth and Genome-Wide Transcriptional Response of the Causal Agent of American Foulbrood Disease (Paenibacillus larvae)
Source: PLoS One. 2014 Feb 20;9(2):e89175. doi: 10.1371/journal.pone.0089175 (PMC3930689; doi:10.1371/journal.pone.0089175)
Supplement: File S1 — Figure S1. Average expression stability (A) and determination of the optimal number (B) of reference targets with geNormPLUS. Figure S2. Validation of microarray data with RTQ-PCR. (A) Log2-transformed expression ratio of T1 compared to C1. (B) Log2-transformed expression ratio of T4 compared to C4. White bars: RTQ-PCR experiment. Black bars: microarray experiment. T1: test sample collected one hour after spiking with larval fluids. T4: test sample collected four hours after spiking with larval fluids. C1: control sample collected one hour after spiking with BHIT-broth. C4: control sample collected four hours after spiking with BHIT-broth. +: differential expression (significant). -: equal expression (non-significant). Figure S3. Validation of microarray data with RTQ-PCR. (A) Log2-transformed expression ratio of C4 compared to C1. (B) Log2-transformed expression ratio of T4 compared to T1. White bars: RTQ-PCR experiment. Black bars: microarray experiment. T1: test sample collected one hour after spiking with hemolymph. T4: test sample collected four hours after spiking with hemolymph. C1: control sample collected one hour after spiking with BHIT-broth. C4: control sample collected four hours after spiking with BHIT-broth. +: differential expression (significant). -: equal expression (non-significant). Figure S4. Stacked percentage bar chart, showing the numbers of up- and down-regulated (putative) transporter encoding genes for T4–T1 (left) and C4–C1 (right), respectively, relative to the total numbers of (putative) transporter encoding genes within the P. larvae genome. The latter are indicated between square brackets. Round brackets: GO term numbers. GO terms were assigned with Blast2GO. White bars: down-regulation for T4–T1. Dark grey bars: up-regulation for T4–T1. Light grey bars: down-regulation for C4–C1. Black bars: up-regulation for C4–C1. Arrow heads: arbitrary GO term hierarchy (;◂>⊲><$>\raster="rg3"<$>). Figure S5. Stacked percentage bar chart, showing t [file pone.0089175.s001.pdf]

Supporting Information

Figure S1. Average expression stability (A) and determination of the optimal number (B) of reference targets with geNorm[PLUS].

(A) Average expression stability of remaining reference targets | **(B) Determination of the optimal number of reference targets**

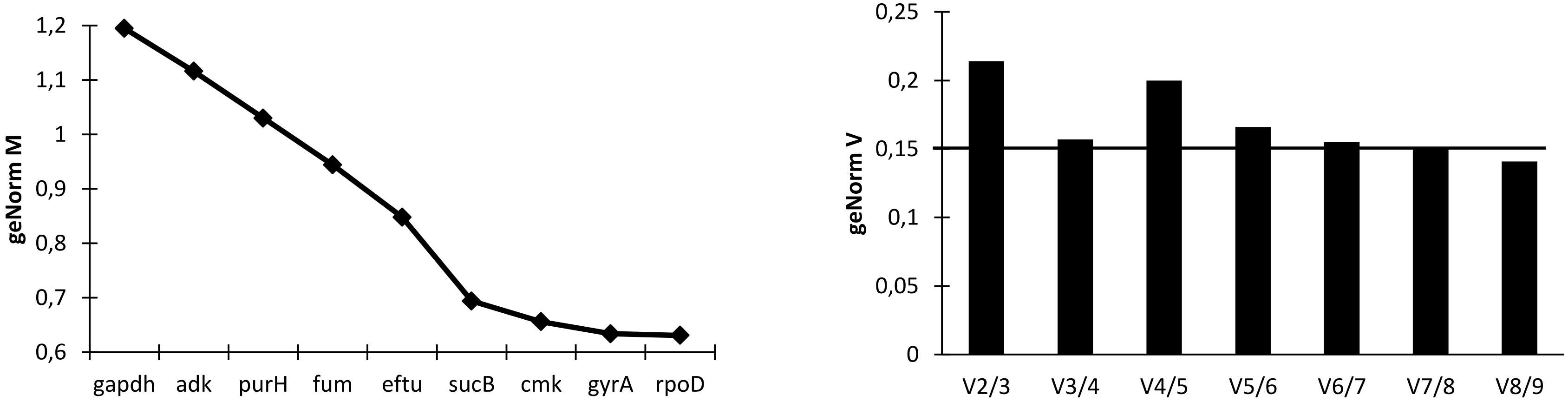

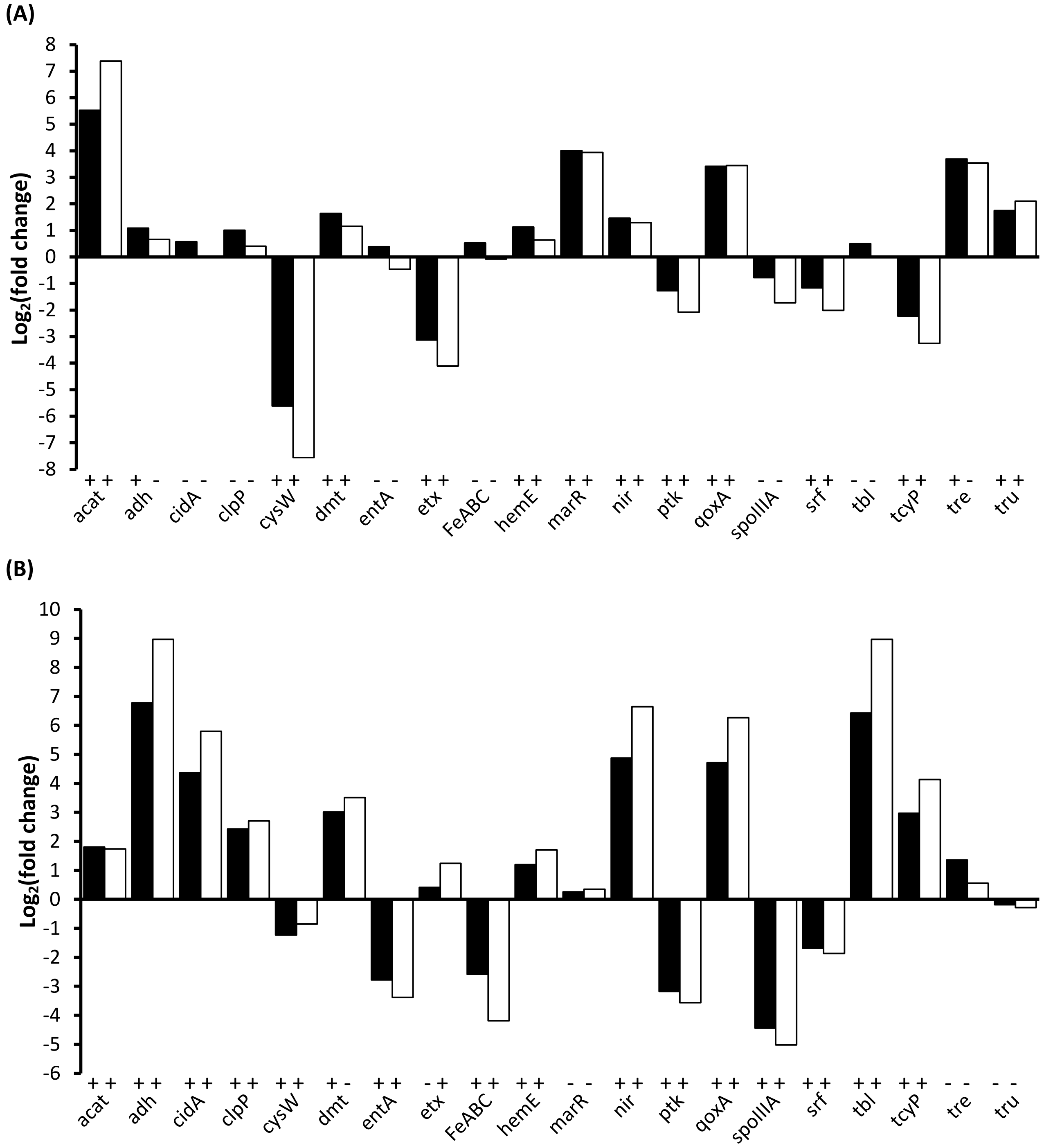

Figure S2. Validation of microarray data with RTQ-PCR. (A) Log_2-transformed expression ratio of T1 compared to C1. (B) Log_2-transformed expression ratio of T4 compared to C4. White bars: RTQ-PCR experiment. Black bars: microarray experiment. T1: test sample collected one hour after spiking with larval fluids. T4: test sample collected four hours after spiking with larval fluids. C1: control sample collected one hour after spiking with BHIT-broth. C4: control sample collected four hours after spiking with BHIT-broth. +: differential expression (significant). -: equal expression (non-significant).

(A)

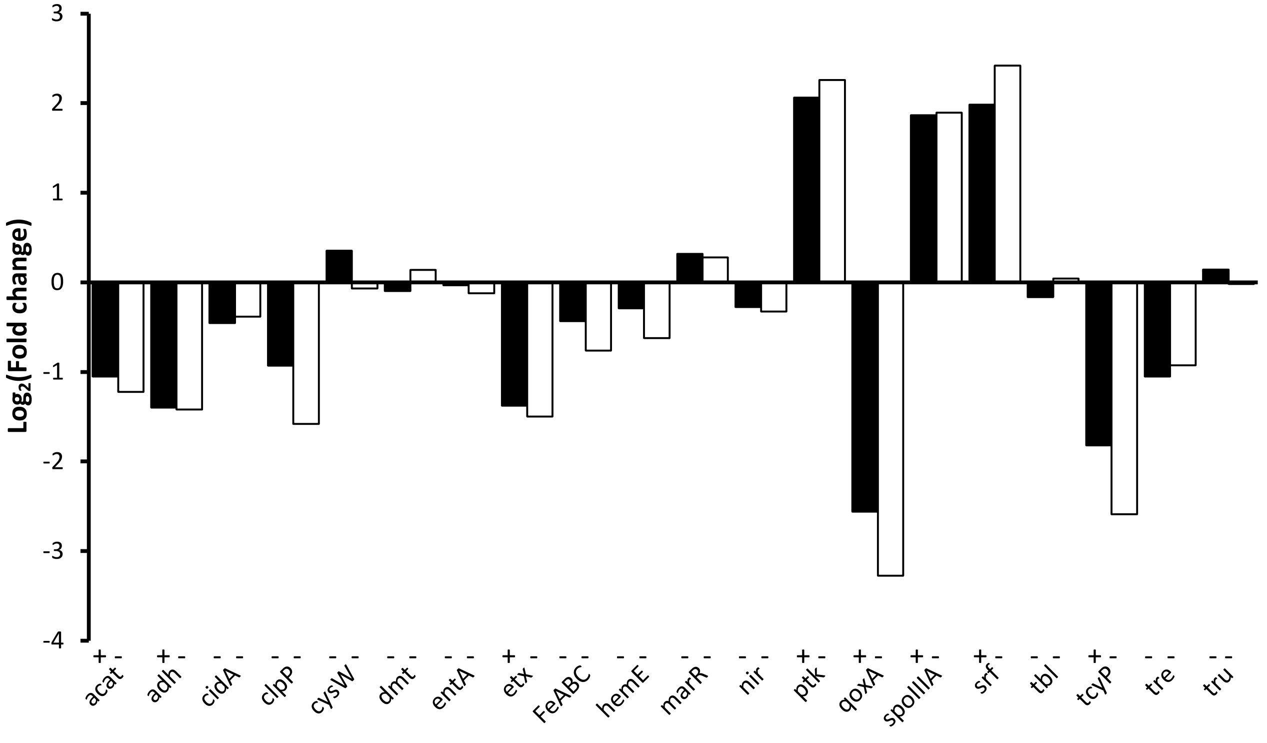

(B)

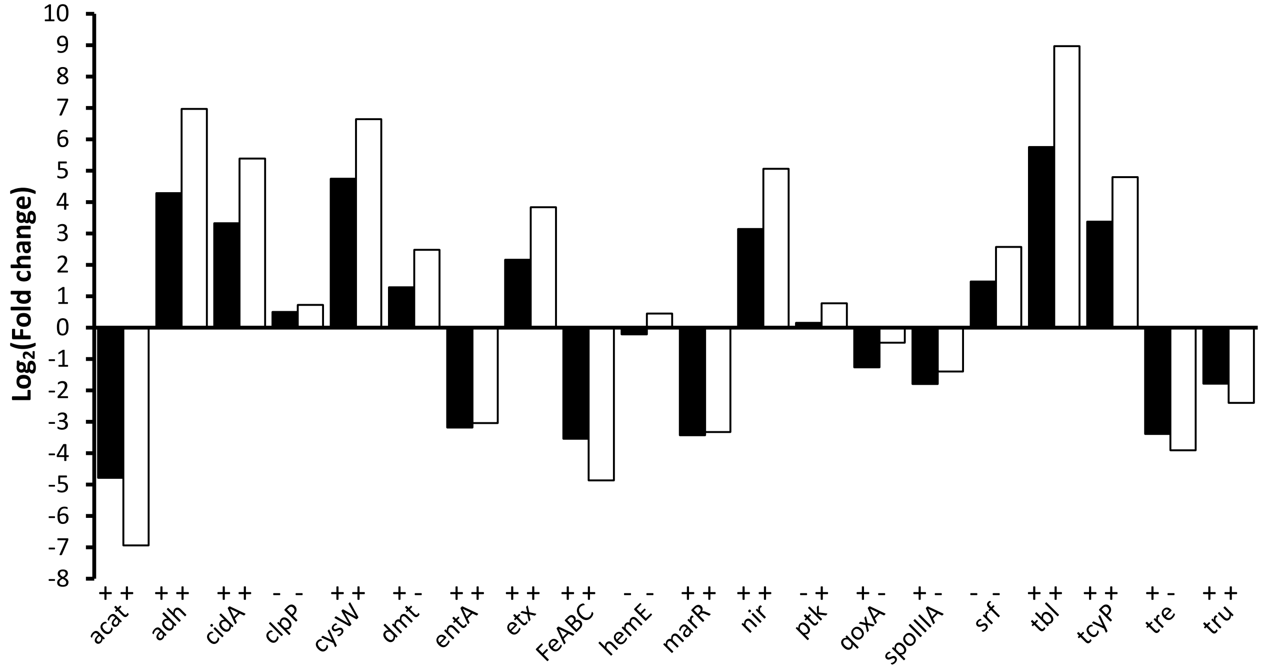

Figure S3. Validation of microarray data with RTQ-PCR. (A) Log_2-transformed expression ratio of C4 compared to C1. (B) Log_2-transformed expression ratio of T4 compared to T1. White bars: RTQ-PCR experiment. Black bars: microarray experiment. T1: test sample collected one hour after spiking with

hemolymph. T4: test sample collected four hours after spiking with hemolymph. C1: control sample collected one hour after spiking with BHIT-broth. C4: control sample collected four hours after spiking with BHIT-broth. +: differential expression (significant). -: equal expression (non-significant).

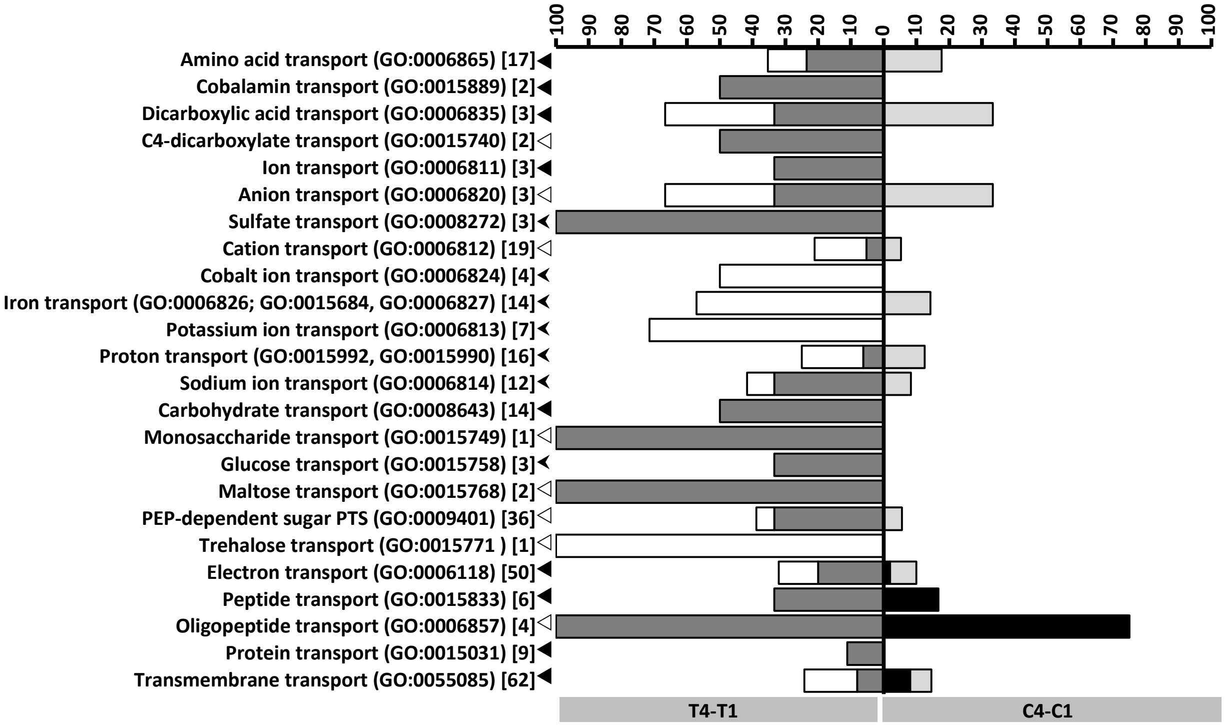

Figure S4. Stacked percentage bar chart, showing the numbers of up- and down-regulated (putative) transporter encoding genes for T4-T1 (left) and C4-C1 (right), respectively, relative to the total numbers of (putative) transporter encoding genes within the *P. larvae* genome. The latter are indicated between square brackets. Round brackets: GO term numbers. GO terms were assigned with Blast2GO. White bars: down-regulation for T4-T1. Dark grey bars: up-regulation for T4-T1. Light grey bars: down-regulation for C4-C1. Black bars: up-regulation for C4-C1. Arrow heads: arbitrary GO term hierarchy (◀ > ◁ > ➤).

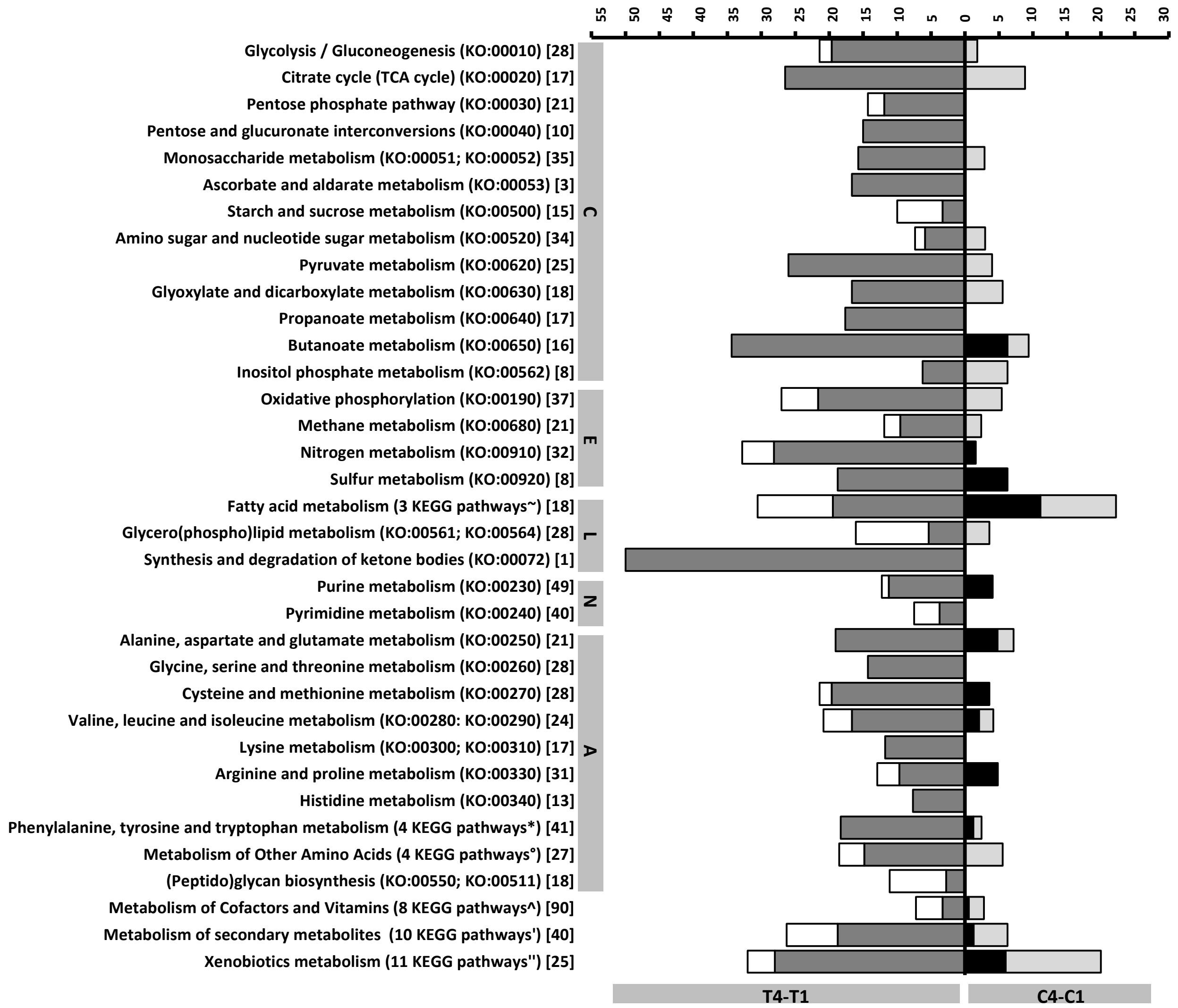

Figure S5. Stacked percentage bar chart, showing the numbers of up- and down-regulated (putative) metabolic genes for T4-T1 (left) and C4-C1 (right), respectively, relative to the total numbers of (putative) metabolic genes within the *P. larvae* genome. C: carbohydrate metabolism. The latter are indicated between square brackets. Round brackets: KO numbers (KEGG pathways). KEGG pathways were assigned with KAAS. White bars: down-regulation for T4-T1. Dark grey bars: up-regulation for T4-T1. Light grey bars: down-regulation for C4-C1. Black bars: up-regulation for C4-C1. ~: KO:00061; KO:00071; KO:00592. *: KO:00350; KO:00360; KO:00380; KO:00400. °: KO:00410; KO:00430; KO:00450; KO:00480. ^: KO:00740; KO:00760; KO:00770; KO:00780; KO:00790; KO:00670; KO:00860; KO:00130. ‘:KO:00900; KO:00903; KO:00281; KO:00523; KO:01053; KO:01055; KO:00960; KO:00232; KO:00521; KO:00401. ‘’: KO:00362; KO:00627; KO:00625; KO:00622; KO:00633; KO:00642; KO:00643; KO:00930; KO:00621; KO:00626; KO:00983

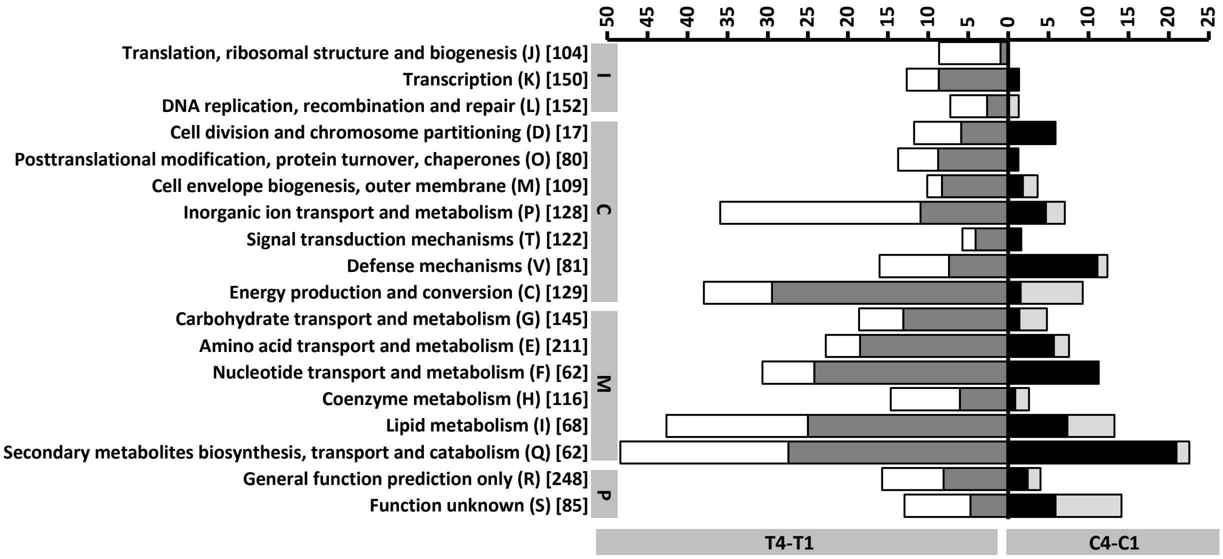

Figure S6. Stacked percentage bar chart, showing the numbers of up- and down-regulated genes for T4-T1 (left) and C4-C1 (right), respectively, relative to the total numbers of genes within the *P. larvae* genome. The latter are indicated between square brackets. Round brackets: COG functional category label. C: cellular processes and signaling. I: information storage and processing. M: metabolism. P: poorly characterized. White bars: down-regulation for T4-T1. Dark grey bars: up-regulation for T4-T1. Light grey bars: down-regulation for C4-C1. Black bars: up-regulation for C4-C1.

Table S1. Reference genes used to normalize the results of the qRT-PCR experiment for microarray data validation.

Reference genes								
Gene name	EC number	Biological process	Primer name	Primer sequence	Amplicon length	Efficiency (%)	R^2	Slope
**RNA polymerase sigma factor RpoD**	-	Transcription	AFB_rpoD_fw<br>AFB_rpoD_rv	AACTTGCCAAACGGATTGAG<br>AAGCCCCATGTTACCTTCCT	149	106.6	0.993	-3.174
**DNA gyrase subunit A**	5.99.1.3	DNA topological change<br>Replication	AFB_gyrA_fw<br>AFB_gyrA_rv	ATGCGGTCATCCCTATTGAG<br>GGTCATCTTCCCGCAAATTA	147	80.8	0.923	-3.887
**Cytidylate kinase**	2.7.4.14	Nucleic acid metabolism<br>Pyrimidine metabolism	AFB_cmk_fw<br>AFB_cmk_rv	GTACAGGGCGATTACCTGGA<br>GCCATCAACGAATACCTGCT	136	92.6	0.994	-3.512
**Succinyl-CoA synthetase subunit beta**	6.2.1.5	Energy metabolism<br>Tricarboxylic acid cycle	AFB_sucB_fw<br>AFB_sucB_rv	ATTGCCAAGGGTGTTGTAGC<br>TTCAGCCCGGATTCATTTAG	119	97.9	0.998	-3.374
**Elongation factor Tu**	-	Translation	AFB_eftu_fw<br>AFB_eftu_rv	TAACATCGGTGCCCTTCTTC<br>CCACCCTCTTCGCTAGTCAG	135	101.2	0.998	-3.294
**Fumarate hydratase**	4.2.1.2	Energy metabolism<br>Tricarboxylic acid cycle	AFB_fum _fw<br>AFB_fum _rv	CCAAAATATGCGGAGCTGAT<br>GTGAACCGCAATTTCCCTTA	144	92.2	0.995	-3.525
**Bifunctional phosphoribosyl aminoimidazole carboxamide formyltransferase IMP cyclohydrolase**	3.5.4.10	Nucleic acid metabolism<br>Purine metabolism	AFB_purH_fw<br>AFB_purH_rv	TTCTCTCGGGGCTTTTGATA<br>CTACTGTTGGCTCACGGTCA	149	105.6	0.995	-3.195
**Adenylate kinase**	2.7.4.3	Nucleic acid metabolism<br>Purine metabolism	AFB_adk_fw<br>AFB_adk_rv	TCAACAGGTGATGCTTTTCG<br>TGTGATTTCGTCAGGAACCA	105	97.8	0.998	-3.377
**Glyceraldehyde-3-phosphate dehydrogenase**	1.2.1.12	Energy metabolism<br>glycolysis/gluconeogenesis	AFB_gapdh_fw<br>AFB_gapdh_rv	TGTTGAAGCTGGTGAAGGTG<br>TCCGCTTTTTCTTTTGCAGT	150	90.1	0.992	-3.585

Table S2. Randomly selected genes used in the RTQ-PCR experiment to validate the microarray results.

Target genes							
**Gene name**	**Biological process**	**Primer name**	**Primer sequence**	**Amplicon length**	**Efficiency (%)**	**R^2**	**Slope**
**alcohol dehydrogenase**	Alcohol metabolism	AFB_adh_fw<br>AFB_adh_rv	TGGCAGTTCAAACCAAAACA<br>GCTTTTTGCGCGTTTTCTAC	82	109.7	0.996	-3.109
**Cytochrome aa3-600 quinol oxidase subunit II**	Oxidative phosphorylation	AFB_qoxA_fw<br>AFB_qoxA_rv	GCTTTTGGAGACGATTTGGA<br>TCCTGGGATTCATCTTTTGG	111	109.1	0.989	-3.122
**Surfactin synthetase**	Antibiotic biosynthesis	AFB_srf_fw<br>AFB_srf_rv	CGAGTTCCCGGTAACACCTA<br>AGATACCGGCCCATATTTCC	134	117.3	0.989	-2.966
**Epsilon toxin**	Pathogenesis	AFB_etx_fw<br>AFB_etx_rv	CAAATCCCTCTTGTGGGAGA<br>TCCACAGACGAATGTGCAG	143	115.5	0.989	-2.999
**Sulfate ABC transporter inner membrane subunit**	Sulfate transport	AFB_cysW_fw<br>AFB_cysW_rv	TGCCGTTTCTCCCGTTATAG<br>CACAAACAAGGTCGCCAGTA	139	118.9	0.990	-3.939
**L-cystine uptake protein**	Amino acid transport	AFB_tcyP_fw<br>AFB_tcyP_rv	ATGCGGAAATGTTCCAGAAG<br>GTCTTGGCGATCGTATTCGT	124	114.6	0.989	-3.015
**iron-siderophore ABC transporter**	Iron transport	AFB_FeABC_fw<br>AFB_FeABC_rv	CCTTGGGGGATCTGGATATT<br>ATTCGGCTGATGCGTATTTC	128	117.5	0.988	-2.963
**α,α-phosphotrehalase**	Carbohydrate metabolism	AFB_tre_fw<br>AFB_tre_rv	CCCTGAGCTTTTCGCTTATG<br>TTTCCAGCATCCAATCCTTC	114	114.4	0.992	-3.020
**assimilatory nitrite reductase large subunit**	Nitrogen metabolism	AFB_nir_fw<br>AFB_nir_rv	CGGTAATAGGAAGCGAACCA<br>TTCCCGTGTGCAGATCAATA	141	117.0	0.985	-2.972
**acetyl-CoA acetyltransferase**	Fatty acid metabolism	AFB_acat_fw<br>AFB_acat_rv	ACGAAAACGGCAAAGTATGG<br>GCTGTAACGCTTCCCTTCAG	127	111.4	0.992	-3.076
**2,3-dihydro-2,3-dihydroxybenzoate dehydrogenase**	Biosynthesis of siderophore group nonribosomal peptides	AFB_entA_fw<br>AFB_entA_rv	TTGGTTTCTCCTGGTTCGAC<br>TATCCGAGGGTAGTGCCAAC	142	106.0	0.994	-3.185
**Stage III sporulation protein AA**	Sporulation	AFB_spoIII_fw	AGGGAAGGAAGACCTCTGGA	138	114.4	0.993	-3.019

		AFB_spoIII_rv	GGTCAGCAGCTCCAGAAAAG				
**Clp protease**	Stress response<br>Degradation of misfolded proteins	AFB_clpP_fw<br>AFB_clpP_rv	CCCAAAGGCTATTTGTTGGA<br>GCTGCGTCTATTGCTTCCAT	100	115.0	0.990	-3.008
**Transcriptional regulator of MarR family**	Transcription	AFB_marR_fw<br>AFB_marR_rv	AAGGGACAGGCAAGACAGAA<br>CTGGTGAAAGCTGCTGCATA	125	115.9	0.994	-2.992
**protein tyrosine kinase**	Signaling	AFB_ptk_fw<br>AFB_ptk_rv	TGCGGACCTTAGAAAGCCTA<br>CATAATGGATGGCGTTTTCC	110	106.0	0.993	-3.187
**tRNA pseudouridine synthase A**	Translation<br>tRNA processing	AFB_tru_fw<br>AFB_tru_rv	CCGGGGACTTATCCAGATTT<br>ACCGGTATTTCCGAGCTCTT	120	118.9	0.994	-2.939
**Thiamine biosynthesis lipoprotein**	Thiamine metabolism	AFB_tbl_fw<br>AFB_tbl_rv	TCGTTTCTTTGTGGGGAATC<br>TACCGGCTTCTTCCAGCTTA	141	116.9	0.994	-2.974
**holin-like protein**	Cytolysis	AFB_cidA_fw<br>AFB_cidA_rv	GGGAGGCCTTGTCTCAGATT<br>CCCTGTTCGAACCATTGAAG	117	117.1	0.991	-2.970
**Drug metabolite exporter**	Drug metabolite transport	AFB_dmt_fw<br>AFB_dmt_rv	AGCAGCTTATAGGCGGTCAA<br>GATCGACGTCACACTCATGG	140	117.7	0.991	-2.960
**Uroporphyrinogen decarboxylase**	Porphyrin metabolism	AFB_hemE_fw<br>AFB_hemE_rv	TGGCTGCAGAAGTAACGATG<br>AAGGAAGCTACCGGGTTCAT	91	109.8	0.995	-3.108

Table S3: Validation of microarray data by qRT-PCR.

Gene	Function	Primers	Slope	R^2	E(%)	T1-C1		T4-C4		C4-C1		T4-T1		
						FC	P	FC	P	FC	P	FC	P	
**METABOLISM**														
**adh**	Alcohol metabolism	F:TGGCAGTTCAAACCAAAACA	-3.109	0.996	109.7	+1.58x	2.86E-01	+500.00x	2.09E-03	-2.67x	6.35E-02	+125.00x	2.27E-04	**Q**
		R:GCTTTTTGCGCGTTTTCTAC				+2.12x	7.81E-03	+108.91x	3.79E-10	-2.63x	3.11E-03	+19.50x	1.16E-07	**M**
**tre**	Carbohydrate metabolism	F:CCCTGAGCTTTTCGCTTATG	-3.020	0.992	114.4	+11.63x	4.57E-01	+1.47x	5.09E-01	-1.90x	6.35E-02	-15.03x	9.01E-02	**Q**
		R:TTTCCAGCATCCAATCCTTC				+12.93x	1.63E-03	+2.57x	2.69E-01	-2.07x	4.36E-01	-10.42x	4.92E-03	**M**
**acat**	Fatty acid metabolism	F:ACGAAAACGGCAAAGTATGG	-3.076	0.992	111.4	+166.67x	4.76E-02	+3.33x	6.61E-03	-2.33x	6.35E-02	-122.30x	4.00E-03	**Q**
		R:GCTGTAACGCTTCCCTTCAG				+46.27x	2.23E-09	+3.49x	7.84E-04	-2.07x	3.36E-02	-27.50x	2.65E-08	**M**
**nir**	Nitrogen metabolism	F:CGGTAATAGGAAGCGAACCA	-2.972	0.985	117.0	+2.44x	4.76E-02	+100.00x	4.42E-04	-1.25x	4.57E-01	+33.33x	1.62E-03	**Q**
		R:TTCCCGTGTGCAGATCAATA				+2.76x	2.57E-06	+29.29x	1.39E-11	-1.21x	2.25E-01	+8.79x	1.40E-09	**M**
**qoxA**	Oxidative phosphorylation	F:GCTTTTGGAGACGATTTGGA	-3.122	0.989	109.1	+10.87x	4.76E-02	+76.92x	5.00E-04	-9.69x	6.35E-02	-1.39x	2.70E-01	**Q**
		R:TCCTGGGATTCATCTTTTGG				+10.68x	2.56E-08	+26.14x	1.97E-09	-5.89x	4.49E-06	-2.41x	1.38E-03	**M**
**hemE**	Porphyrin metabolism	F:TGGCTGCAGAAGTAACGATG	-3.108	0.995	109.8	+1.56x	4.76E-02	+3.26x	4.96E-02	-1.54x	6.35E-02	+1.36x	3.58E-01	**Q**
		R:AAGGAAGCTACCGGGTTCAT				+2.18x	3.02E-	+2.29x	9.41E-	-1.22x	2.20E-01	-1.16x	2.95E-	**M**

							05		05				01	
**tbl**	Thiamine metabolism	F:TCGTTTCTTTGTGGGGAATC	-2.974	0.994	116.9	+1.02x	7.22E-01	+500.00x	1.22E-06	+1.03x	6.07E-01	+500.00x	1.14E-06	**Q**
		R:TACCGGCTTCTTCCAGCTTA				+1.42x	6.47E-02	+85.80x	9.84E-12	-1.12x	8.81E-01	+54.08x	2.72E-11	**M**
**Transport**														
**tcyP**	Amino acid transport	F:ATGCGGAAATGTTCCAGAAG	-3.015	0.989	114.6	-9.57x	4.76E-02	+17.54x	6.61E-03	-6.01x	6.35E-02	+27.78x	4.37E-03	**Q**
		R:GTCTTGGCGATCGTATTCGT				-4.69x	4.38E-05	+7.81x	1.15E-05	-3.53x	8.07E-04	+10.37x	1.60E-06	**M**
**dmt**	Drug transport	F:AGCAGCTTATAGGCGGTCAA	-2.960	0.991	117.7	+2.23x	4.76E-02	+11.36x	6.02E-02	+1.10x	7.62E-01	+5.59x	1.10E-01	**Q**
		R:GATCGACGTCACACTCATGG				+3.11x	1.12E-03	+8.08x	1.62E-05	-1.07x	9.06E-01	+2.43x	2.64E-02	**M**
**FeABC**	Iron transport	F:CCTTGGGGGATCTGGATATT	-2.963	0.988	117.5	-1.06x	8.86E-01	-18.22x	3.46E-02	-1.70x	4.57E-01	-29.25x	1.31E-02	**Q**
		R:ATTCGGCTGATGCGTATTTC				+1.44x	6.08E-01	-6.00x	1.32E-02	-1.35x	7.58E-01	-11.66x	9.42E-04	**M**
**cysW**	Sulphate transport	F:TGCCGTTTCTCCCGTTATAG	-3.939	0.990	118.9	-188.13x	4.76E-02	-1.81x	3.46E-02	-1.05x	1.00E+00	+100.00x	2.27E-04	**Q**
		R:CACAAACAAGGTCGCCAGTA				-48.94x	6.80E-10	-2.34x	5.99E-03	+1.28x	4.50E-01	+26.69x	1.72E-08	**M**

Continued on the next page

Table S4: Validation of microarray data by qRT-PCR.

Gene	Function	Primers	Slope	R^2	E(%)	T1-C1		T4-C4		C4-C1		T4-T1		
						FC	P	FC	P	FC	P	FC	P	
**OTHER CATEGORIES**														
**srf**	Antibiotic biosynthesis	F:CGAGTTCCCGGTAACACCTA	-2.966	0.989	117.3	-4.04x	4.76E-02	-3.64x	2.35E-03	+5.35x	6.35E-02	+5.95x	3.68E-03	**Q**
		R:AGATACCGGCCCATATTTCC				-2.24x	7.72E-04	-3.21x	8.04E-05	+3.95x	1.76E-05	+2.76x	2.11E-04	**M**
**cidA**	Cytolysis	F:GGGAGGCCTTGTCTCAGATT	-2.970	0.991	117.1	+1.02x	7.22E-01	+55.56x	2.66E-03	-1.31x	3.64E-01	+41.67x	4.00E-03	**Q**
		R:CCCTGTTCGAACCATTGAAG				+1.49x	1.48E-01	+20.52x	5.73E-09	-1.37x	1.69E-01	+10.05x	7.92E-08	**M**
**entA**	Siderophore group NRPS	F:TTGGTTTCTCCTGGTTCGAC	-3.185	0.994	106.0	-1.38x	7.22E-01	-10.44x	1.09E-02	-1.09x	1.00E+00	-8.23x	8.23E-03	**Q**
		R:TATCCGAGGGTAGTGCCAAC				+1.30x	4.90E-01	-6.85x	1.86E-04	-1.02x	9.64E-01	-9.11x	3.09E-05	**M**
**etx**	Pathogenesis	F:CAAATCCCTCTTGTGGGAGA	-2.999	0.989	115.5	-17.26x	4.76E-02	+2.36x	4.46E-02	-2.83x	6.35E-02	+14.29x	1.31E-02	**Q**
		R:TCCACAGACGAATGTGCAG				-8.75x	1.06E-06	+1.33x	3.55E-01	-2.60x	4.36E-03	+4.47x	1.02E-04	**M**

ptk	Signaling	F:TGCGGACCTTAGAAAGCCTA	-3.187	0.993	106.0	-4.23x	4.76E-02	-11.83x	1.45E-03	+4.78x	3.64E-01	+1.71x	4.00E-03	Q
		R:CATAATGGATGGCGTTTTCC				-2.41x	2.72E-02	-9.06x	1.32E-04	+4.17x	4.42E-03	+1.11x	8.82E-01	M
spoIIAA	Sporulation	F:AGGGAAGGAAGACCTCTGGA	-3.019	0.993	114.4	-3.31x	1.76E-01	-32.49x	2.66E-03	+3.72x	4.57E-01	-2.64x	9.05E-02	Q
		R:GGTCAGCAGCTCCAGAAAAG				-1.72x	1.04E-01	-21.71x	7.26E-07	+3.65x	1.86E-03	-3.47x	2.13E-03	M
clpP	Stress	F:CCCAAAGGCTATTTGTTGGA	-3.008	0.990	115.0	+1.33x	6.07E-01	+6.54x	6.61E-03	-2.99x	6.35E-02	+1.65x	1.54E-01	Q
		R:GCTGCGTCTATTGCTTCCAT				+2.00x	1.32E-01	+5.38x	1.63E-03	-1.90x	1.87E-01	+1.41x	4.78E-01	M
marR	Transcription	F:AAGGGACAGGCAAGACAGAA	-2.992	0.994	115.9	+15.38x	4.76E-02	+1.27x	3.56E-01	+1.21x	4.57E-01	-9.99x	4.00E-03	Q
		R:CTGGTGAAAGCTGCTGCATA				+16.07x	3.07E-09	+1.20x	4.79E-01	+1.25x	4.55E-01	-10.77x	9.32E-08	M
tru	Translation tRNA processing	F:CCGGGGACTTATCCAGATTT	-2.939	0.994	118.9	+4.29x	4.76E-02	-1.22x	3.68E-01	-1.01x	7.62E-01	-5.27x	3.05E-03	Q
		R:ACCGGTATTTCCGAGCTCTT				+3.35x	2.32E-06	-1.14x	7.20E-01	+1.10x	6.66E-01	-3.45x	1.40E-05	M

Sequences (F: forward; R: reverse) and efficiencies (E) of the primers for the twenty validated genes are displayed. The primer efficiencies (E) were assessed by a standard dilution series of pooled cDNA (five-fold dilutions, five different dilutions, three replicates for each dilution). The values for

slope and coefficient (R^2) of the linear regression trendline are also given. The last columns mention the fold change of expression (FC) and p-value (P) for the twenty validated genes, as determined by qRT-PCR (Q) and microarray (M). Four comparisons were made (T1-C1, T4-C4, C4-C1, T4-T1). +(green cells): up-regulation. - (red cells): down-regulation. Dark green/red cells: differential expression (significant). Light green/red cells: equal expression (non-significant).

Table S5: GO-enrichment analysis for comparison C4-C1, showing the most specific over- and under-represented biological process GO-terms for both up- and down-regulation.

Biological process	GO number	NS	FE	P-value
**Up-regulation**				
**Over-representation**				
De novo IMP biosynthetic process	GO:0006189	4	45.63	2.50E-05
Oligopeptide transport	GO:0006857	3	101.0	1.00E-04
**Under-representation**				
Cellular macromolecule metabolic process	GO:0044260	3	0.14	1.40E-05
**Down-regulation**				
**Over-representation**				
ATP synthesis coupled electron transport	GO:0042773	4	24.98	8.10E-05
Biotin biosynthetic process	GO:0009102	4	∞	1.90E-07
Cation transport	GO:0006812	7	5.93	5.10E-04
Electron transport	GO:0006118	7	8.64	6.40E-05
Polyol metabolic process	GO:0019751	4	11.70	8.50E-04
Reductive tricarboxylic acid cycle	GO:0019643	4	19.96	1.60E-04
**Under-representation**				
Biological regulation	GO:0065007	0	0	3.80E-04
Cellular macromolecule metabolic process	GO:0044260	0	0	2.00E-06
Nucleic acid metabolic process	GO:0090304	0	0	7.70E-05

NS, FE, P-value: see table X

Table S6: GO-enrichment analysis for comparison T4-T1, showing the most specific over- and under-represented biological process GO-terms for both up- and down-regulation.

Biological process	GO number	NS	FE	P-value
**Up-regulation**				
**Over-representation**				
Carbohydrate transport	GO:0008643	15	5.39	6.40E-06
De novo IMP biosynthetic process	GO:0006189	6	46.16	1.60E-05
Glyoxylate cycle	GO:0006097	3	∞	1.60E-03
Limonene catabolic process	GO:0046251	5	∞	2.20E-05
Lysine catabolic process	GO:0006554	5	∞	2.20E-05
Mitochondrial electron transport, NADH to ubiquinone	GO:0006120	5	∞	2.20E-05
Nitrate assimilation	GO:0042128	3	∞	1.60E-03
Oligopeptide transport	GO:0006857	4	∞	1.90E-04
Photosynthesis, light reaction	GO:0019684	5	∞	2.20E-05
Sodium ion transport	GO:0006814	6	7.67	1.20E-03
Sulfate transport	GO:0008272	3	∞	1.60E-03
Sulfur amino acid biosynthetic process	GO:0000097	7	5.98	1.30E-03
Tricarboxylic acid cycle	GO:0006099	9	5.36	4.60E-04
Ubiquinone biosynthetic process	GO:0006744	5	19.14	3.70E-04
β-alanine metabolic process	GO:0019482	5	12.75	9.00E-04
**Under-representation**				
Biological regulation	GO:0065007	23	0.48	6.90E-04
DNA metabolic process	GO:0006259	2	0.09	1.30E-06
Ribosome biogenesis	GO:0042254	0	0	2.00E-03
RNA metabolic process	GO:0016070	8	0.31	3.20E-04
Translation	GO:0006412	0	0	1.60E-05
**Down-regulation**				
**Over-representation**				
High-affinity iron ion transport	GO:0006827	4	∞	3.80E-05
Potassium ion transport	GO:0006813	5	30.03	5.40E-05
Siderophore biosynthetic process	GO:0019290	4	∞	3.80E-05
**Under-representation**				
Cellular nitrogen compound metabolic process	GO:0034641	38	0.52	4.40E-04
Organic acid metabolic process	GO:0006082	9	0.35	8.00E-04

NS, FE, P-value: see table X

Table S7: Annotation and pathway analysis for both up- and down-regulated genes for C4-C1.

Pathway	MetaCyc ID	
**Up-regulation**		
5-aminoimidazole ribonucleotide biosynthesis I	PWY-6121	2/8
5-aminoimidazole ribonucleotide biosynthesis II	PWY-6122	2/8
Adenine and adenosine salvage IV	PWY-6610	1/5
Arginine degradation VII	ARG-GLU-PWY	1/2
Asparagine biosynthesis I	ASPARAGINE-BIOSYNTHESIS	1/3
Citrulline biosynthesis	CITRULBIO-PWY	1/9
Cysteine biosynthesis I	CYSTSYN-PWY	1/5
Fatty acid biosynthesis initiation I	PWY-4381	1/7
Guanine and guanosine salvage I	PWY-6620	1/4
Methylthiopropionate biosynthesis	PWY-5389	1/1
Ornithine degradation I	ORN-AMINOPENTANOATE-CAT-PWY	1/1
Xanthine and xanthosine salvage	SALVPURINE2-PWY	1/3
**Down-regulation**		
Aspartate degradation II	MALATE-ASPARTATE-SHUTTLE-PWY	1/2
Biotin biosynthesis from 7-keto-8-aminopelargonate	PWY0-1507	4/4
dTDP-L-rhamnose biosynthesis I	DTDPRHAMSYN-PWY	1/7
Selenocysteine biosynthesis I	PWY0-901	1/4

ID: MetaCyc identifier. #: number of up- or down-regulated genes annotated with a particular pathway in relation to the number of genes annotated with that pathway.

Table S8: Annotation and pathway analysis for both up- and down-regulated genes for T4-T1.

Pathway	MetaCyc ID	#
**Down-regulation**		
2,3-dihydroxybenzoate biosynthesis	PWY-5901	3/3
Arginine degradation III	PWY0-823	1/2
Arginine dependent acid resistance	PWY0-1299	1/1
Asparagine degradation I	ASPARAGINE-DEG1-PWY	1/1
CDP-diacylglycerol biosynthesis I	PWY-5667	1/5
CDP-diacylglycerol biosynthesis II	PWY0-1319	1/5
Demethylmenaquinol-8 biosynthesis I	PWY-5852	1/1
Glycerol degradation I	PWY0-381	2/3
Glycerol degradation IV	PWY-4261	2/2
Leucine biosynthesis	LEUSYN-PWY	1/7
Nitrogen fixation	N2FIX-PWY	1/1
Pentose phosphate pathway	OXIDATIVEPENT-PWY	1/4
Trehalose degradation I	TREDEGLOW-PWY	1/4

(Continued on the next page)

Table S8: Annotation and pathway analysis for both up- and down-regulated genes for T4-T1.

Pathway	MetaCyc ID	
**Up-regulation**		
5-aminoimidazole ribonucleotide biosynthesis I	PWY-6121	5/8
5-aminoimidazole ribonucleotide biosynthesis II	PWY-6122	5/8
5-dehydro-4-deoxy-D-glucuronate degradation	PWY-6507	2/4
acetate conversion to acetyl-CoA	PWY0-1313	1/1
Acetyl-CoA biosynthesis	PYRUVDEHYD-PWY	3/9
adenine and adenosine salvage IV	PWY-6610	1/5
Alkylnitronates degradation	PWY-723	3/4
Arginine degradation VII	ARG-GLU-PWY	1/2
Aspartate degradation II	MALATE-ASPARTATE-SHUTTLE-PWY	1/2
Branched-chain alpha-keto acid dehydrogenase complex	PWY-5046	1/6
Cadmium transport I	PWY-6213	1/2
Citrulline biosynthesis	CITRULBIO-PWY	1/9
Cysteine biosynthesis I	CYSTSYN-PWY	2/5
dTDP-L-rhamnose biosynthesis I	DTDPRHAMSYN-PWY	2/7
Ethanol degradation II (cytosol)	PWY66-21	1/4
Ethanol degradation IV	PWY66-162	1/4
Fatty acid biosynthesis initiation I	PWY-4381	1/7
Glutamate biosynthesis I	GLUTSYN-PWY	1/1
Glutamate biosynthesis IV	GLUGLNSYN-PWY	1/1
Glutamine degradation II	GLUTAMINEFUM-PWY	1/1
Glycine betaine biosynthesis II	PWY-3722	1/2
Glycine cleavage complex	GLYCLEAV-PWY	3/6
Guanine and guanosine salvage I	PWY-6620	1/4
Lysine biosynthesis III	PWY-2942	1/7
Lysine biosynthesis VI	PWY-5097	1/9
Methionine biosynthesis I	HOMOSER-METSYN-PWY	1/7
Methylthiopropionate biosynthesis	PWY-5389	1/1
NADH to cytochrome bd oxidase electron transfer	PWY0-1334	10/11
Ornithine degradation I	ORN-AMINOPENTANOATE-CAT-PWY	1/1
Pentose phosphate pathway	NONOXIPENT-PWY	1/5
Pyruvate fermentation to ethanol I	PWY-5480	1/4
Pyruvate fermentation to ethanol III	PWY-6587	1/5
Reductive monocarboxylic acid cycle	PWY-5493	2/3
Seed germination protein turnover	PWY-6018	1/3
UDP-N-acetylmuramoyl-pentapeptide biosynthesis III	PWY-6387	1/15
Wound-induced proteolysis I	PWY-5988	1/3
Xanthine and xanthosine salvage	SALVPURINE2-PWY	1/3
